# Exploratory Identification of Potential Serum Biomarkers Associated with Relapse in Multiple Myeloma

**DOI:** 10.3390/diseases14100380

**Published:** 2026-10-16

**Authors:** Wittawat Chantkran, Chonlada Laoruangroj, Sittiruk Roytrakul, Oytip Nathalang, Pasra Arnutti, Janeyuth Chaisakul, Dollapak Apipongrat

**Affiliations:** 1 Department of Pathology, Phramongkutklao College of Medicine, Bangkok 10400, Thailand; chantkran@yahoo.com; 2 Division of Hematology, Department of Medicine, Phramongkutklao Hospital, Bangkok 10400, Thailand; amchon25@gmail.com; 3 Proteomics Research Laboratory, National Center for Genetic Engineering and Biotechnology, Pathumthani 12120, Thailand; sittiruk@biotec.or.th; 4 Graduate Program in Biomedical Sciences, Faculty of Allied Health Sciences, Thammasat University, Pathumthani 12120, Thailand; oytipntl@hotmail.com; 5 Department of Medical Technology, Faculty of Allied Health Sciences, Pathumthani University, Pathumthani 12000, Thailand; pasra@hotmail.com; 6 Department of Pharmacology, Phramongkutklao College of Medicine, Bangkok 10400, Thailand; janeyuth.cha@pcm.ac.th

**Keywords:** serum biomarkers, multiple myeloma, relapsed and/or refractory, proteomics, CD30

## Abstract

**Background:** Relapse remains a major clinical challenge in multiple myeloma (MM), highlighting the need to identify circulating proteins associated with relapse as candidates for disease monitoring and further biomarker development. **Methods:** An integrated proteomic and transcriptomic approach was used to identify candidate biomarkers associated with relapsed MM. Differentially abundant proteins were identified by LC–MS/MS profiling of paired serum samples collected at very good partial response (≥VGPR) and at relapsed/refractory MM (RRMM) diagnosis. A publicly available gene expression dataset (GSE47552) comparing malignant and normal plasma cells was analyzed to assess gene–protein concordance. Candidate biomarkers were subsequently evaluated by ELISA using paired serum samples from 40 patients collected at ≥VGPR and relapse. Associations with bone marrow plasma cell (BMPC) burden and CD30 expression by immunohistochemistry (IHC) were further explored. **Results:** LC–MS/MS profiling identified 938 proteins, including 84 increased and 39 decreased in RRMM compared with ≥VGPR. Based on predefined selection criteria, TNFRSF8 (CD30) and PTBP2 were prioritized as candidate biomarkers. Serum TNFRSF8 levels were significantly higher at relapse than at ≥VGPR (*p* < 0.001), whereas PTBP2 levels showed no significant change (*p* = 0.578). Serum TNFRSF8 levels were not associated with BMPC burden, and CD30 was absent on plasma cells by IHC, suggesting an increase independent of plasma cell expansion. **Conclusions:** This exploratory study identified serum TNFRSF8 as a candidate circulating biomarker associated with relapse in MM. Further mechanistic and clinical studies in larger, well-characterized prospective cohorts are warranted to clarify its biological basis and potential clinical relevance.

## 1. Introduction

Multiple myeloma (MM) is an incurable malignancy of plasma cells and represents the second most common hematological malignancy, accounting for approximately 1–2% of all cancer cases [1,2]. According to the International Myeloma Working Group (IMWG) criteria, the disease is characterized by the clonal proliferation of malignant plasma cells within the bone marrow (BM), leading to end-organ damage—commonly manifested as osteolytic bone lesions, renal impairment, anemia, and hypercalcemia—and other specific myeloma-defining events (MDEs) [1,2,3,4]. The incidence of MM ranges from 1.5 to 6.0 per 100,000 person-years and is strongly influenced by age and sex [5,6]. More than 50% of patients are diagnosed after the age of 65 years, with a median age at diagnosis of approximately 69 years [2,5,6]. Over recent decades, the global incidence of MM has more than doubled and is projected to continue increasing, largely attributable to population aging [7]. Despite substantial advances in therapeutic strategies—including proteasome inhibitors, immunomodulatory agents, monoclonal antibodies, chimeric antigen receptor (CAR) T-cell therapies, and autologous stem cell transplantation (ASCT)—that have significantly improved response rates and prolonged survival, most patients eventually experience relapse or develop treatment resistance [8,9,10,11]. Moreover, the biological mechanisms underlying disease relapse and therapeutic resistance remain incompletely understood [8,9,10,11].

Relapsed and/or refractory multiple myeloma (RRMM) is an aggressive form of the disease that is difficult to treat and is associated with unfavorable outcomes [12,13]. RRMM is defined by disease progression despite ongoing therapy, progression within 60 days following the last treatment after achieving remission, failure to achieve at least a minimal response (MR), or the presence of primary refractory MM [3,4,14,15]. The management of RRMM has become increasingly complex, posing substantial challenges for clinicians in selecting optimal therapeutic strategies [8,10,12,13]. Consequently, there is a critical need for novel biomarkers capable of accurately predicting the early onset of relapse or refractory disease; however, the identification of such biomarkers remains a significant challenge [16,17].

In our previous study, we employed a proteomic approach to identify potential prognostic biomarkers associated with disease activity and predictive of unfavorable outcomes in the sera of patients with MM [18]. In the present study, we reanalyzed these serum proteomic data to identify circulating proteins associated with relapse by comparing responder and RRMM cohorts. Candidate proteins were subsequently evaluated in paired clinical samples, and their associations with disease status and tumor burden were further explored.

## 2. Materials and Methods

### 2.1. Acquisition of Proteomic Profiles and Identification of Signature Proteins in RRMM

Serum proteomic profiles were generated for patients with RRMM (*n* = 90), MM patients who achieved at least a very good partial response (≥VGPR) to treatment (*n* = 228), newly diagnosed MM patients (*n* = 57), and age-matched unrelated healthy controls (*n* = 70) using liquid chromatography–tandem mass spectrometry (LC–MS/MS), as previously described [18]. In the present study, these proteomic datasets were reanalyzed to identify signature proteins specifically associated with RRMM, with a focused comparison between RRMM patients and MM patients who achieved ≥VGPR. Proteins exhibiting a Log_2_ fold change (RRMM vs. ≥VGPR) greater than 1.5 or less than −1.5, together with adjusted *p*-values < 0.05, were considered significantly differentially expressed.

### 2.2. Protein Functional and Pathway Analyses

To explore the potential functions and pathways associated with differentially expressed proteins, Protein Analysis Through Evolutionary Relationships (PANTHER, version 11.1; available at http://www.pantherdb.org, accessed on 15 August 2025) was used, with *Homo sapiens* selected as the reference organism [19]. Gene Ontology (GO) functional annotation was performed according to molecular function (MF), biological process (BP), and cellular component (CC) categories. To further evaluate the functional roles and pathway involvement of RRMM signature proteins, Kyoto Encyclopedia of Genes and Genomes (KEGG) pathway enrichment analyses were performed using the STRING database (available at https://string-db.org, accessed on 15 August 2025). GO terms and KEGG pathways were considered significantly enriched at a false discovery rate (FDR) < 0.01.

### 2.3. Analysis of Gene Expression Using Publicly Available Datasets

In parallel, we conducted an independent analysis using publicly available gene expression profiles (GEPs) to investigate the potential involvement of the identified signature proteins in MM. Gene expression data were retrieved from the National Center for Biotechnology Information (NCBI) Gene Expression Omnibus (GEO) microarray dataset (accession number GSE47552) [20], which compares gene expression profiles of plasma cells isolated from MM patients and normal controls. The data of genes encoding the candidate signature proteins, including top 20 upregulated and top 20 downregulated proteins, were extracted and comparatively analyzed. Genes exhibiting a significant difference in mean expression between MM patients and normal controls, with a *p*-value < 0.05, were considered differentially expressed.

### 2.4. Transcriptome-Assisted Candidate Prioritization

**Figure 1:**
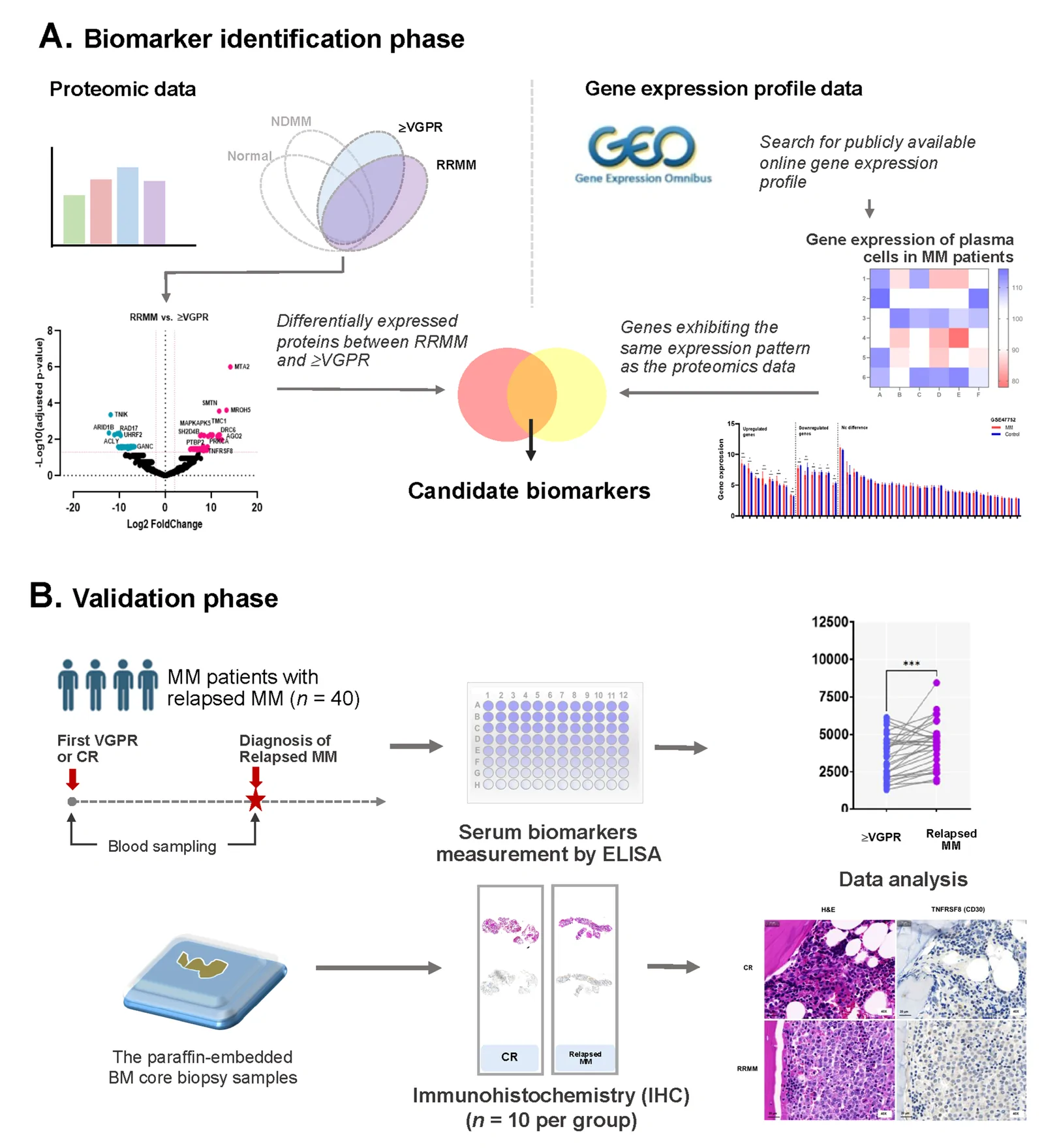
Schematic workflow illustrating the overall study design. (**A**) Biomarker identification strategy integrating LC–MS/MS–based proteomic analysis with corresponding gene expression profiling data. (**B**) Validation cohort design using paired serum samples from patients with relapsed MM to confirm serum levels of candidate biomarkers; tissue expression was assessed by immunohistochemistry (IHC).

The overall biomarker identification strategy is illustrated in Figure 1A. Transcriptomic data were used as an orthogonal, hypothesis-generating approach to prioritize candidate proteins identified by serum proteomics. The analysis comprised two sequential steps: (1) identification of proteins with differential serum abundance between patients with RRMM and those who had achieved ≥VGPR in the LC–MS/MS dataset, and (2) evaluation of the corresponding genes for differential expression between myeloma and normal plasma cells in the GSE47552 dataset. Proteins that met the specified criteria in both datasets and exhibited concordant directions of change were prioritized for further evaluation. This cross-platform concordance was considered supporting evidence of potential disease-related biological relevance rather than direct validation of the serum biomarkers.

Candidates were retained if they fulfilled all of the following criteria: (i) significant differential serum abundance between the RRMM and ≥VGPR groups (*p* < 0.05), with an absolute log_2_ fold change greater than 5.0 in the LC–MS/MS dataset [18]; (ii) significant differential expression of the corresponding gene between myeloma and normal plasma cells (*p* < 0.05), with a fold change greater than 1.2 for upregulation or less than 0.83 for downregulation in the GSE47552 dataset [20]; and (iii) concordant directions of change in the proteomic and transcriptomic datasets. Proteins that failed to meet any of these criteria were excluded.

### 2.5. Validation Cohort of Relapsed MM Patients

The validation cohort comprised 40 unique patients with relapsed MM, and each patient contributed two paired serum samples, resulting in a total of 80 serum samples for validation. The paired samples were collected at two prespecified clinical time points: (1) when patients first achieved a treatment response of ≥VGPR and (2) at the time of diagnosis of relapsed MM. None of the patients in the validation cohort overlapped with the patients whose samples were included in the proteomic discovery dataset, thereby ensuring the independence of the discovery and validation cohorts.

In the validation cohort, serum samples were obtained from patients with relapsed MM who were followed at the Division of Hematology, Department of Medicine, Phramongkutklao Hospital, Bangkok, Thailand, between February 2024 and December 2025. Patients were eligible for inclusion based on the availability of paired serum samples at the two prespecified time points. Samples with insufficient volume (<1.0 mL) or incomplete clinical information in the medical records were excluded. All serum samples were retrieved from the local specimen bank and had been collected as leftover specimens from routine serum protein electrophoresis (SPEP) analyses, aliquoted, and stored at −80 °C until analysis. The paired samples were used to validate serum TNFRSF8 levels, as illustrated in Figure 1B.

To minimize pre-analytical variability, all routine SPEP leftover samples at our center are processed according to a standardized biorepository protocol. Briefly, serum is separated within 4 h of blood collection, aliquoted immediately to avoid repeated freeze–thaw cycles, and archived at −80 °C. Only primary aliquots that had been thawed exactly once specifically for the present enzyme-linked immunosorbent assay (ELISA) measurements were used. No specimens that had undergone previous freeze–thaw cycles were included in this study.

Baseline clinical characteristics, including age at diagnosis, sex, MM isotype, and disease stage according to the International Staging System (ISS) [21], were obtained through retrospective review of medical records. Clinical outcomes, including disease progression, relapse/refractory status, and mortality, were documented through prospective follow-up. Treatment responses were assessed according to the IMWG response criteria [3,4]. Data from medical records were accessed in October 2025 for research purposes. Identifiable participant information (e.g., hospital identification numbers) was accessible during data collection. All data were de-identified prior to analysis to ensure participant confidentiality.

### 2.6. Enzyme-Linked Immunosorbent Assay (ELISA)

The serum concentrations of tumor necrosis factor receptor superfamily member 8 (TNFRSF8) and polypyrimidine tract-binding protein 2 (PTBP2) were measured using commercial ELISA kits; PicoKine^TM^ ELISA for Human TNFRSF8 or CD30 (catalog No. MBS177619, MyBioSource, San Diego, CA, USA) and Human PTBP2 ELISA kit (catalog No. MBS910054 MyBioSource, San Diego, CA, USA). The assay procedure was performed according to the manufacturer’s instructions. Each protein concentration was measured in duplicate using a microplate reader (Synergy^TM^ HT, BioTek Instrument, Winooski, VT, USA).

### 2.7. Immunohistochemistry (IHC) Staining

The paraffin-embedded BM core biopsies obtained from 10 patients with MM, collecting at complete response (CR) and at diagnosis of relapsed MM, were analyzed for the protein expression of TNFRSF8 (CD30). IHC staining was performed on a Ventana Discovery XT automated system (Ventana Medical System, Tucson, AZ, USA), using the primary antibody raised against TNFRSF8 (diluted 1:200; rabbit Anti-human CD30 antibody [EPR4102], ab134080, Abcam, Cambridge, UK), as described in the previous study [18]. Normal tonsil tissue was used as the CD30-positive control in each IHC staining slide [22]. Two slides from each biopsy were stained with hematoxylin and eosin (H&E) for routine histological evaluation. The IHC result was evaluated by two independent pathologists and an average percentage of positive-staining cells was calculated.

### 2.8. Statistical Analysis

Continuous variables were summarized as mean, median, SD, and/or interquartile range (IQR), as appropriate, and were compared using Student’s *t*-test, paired *t*-test, Mann–Whitney U test, Kruskal–Wallis test, Wilcoxon signed-rank test, or analysis of variance (ANOVA), as appropriate. The magnitude of within-patient changes was quantified using the Hodges–Lehmann estimate with its corresponding 95% confidence interval (CI). Simple linear regression was used to assess linear relationships between continuous variables. Categorical variables were described as frequency and percentage. Clinical data and laboratory parameters were compared among diverse groups using Chi-Square (*χ*^2^) or Fisher’s exact tests. Statistical analysis was performed using IBM SPSS Statistics for Windows, Version 20.0 (IBM Corp., Armonk, NY, USA) and GraphPad Prism, Version 9 (GraphPad Software, Boston, MA, USA). A *p*-value of less than 0.05 was considered statistically significant.

## 3. Results

### 3.1. Proteomic Profiling and Functional Enrichment of RRMM Signature Proteins

**Figure 2:**
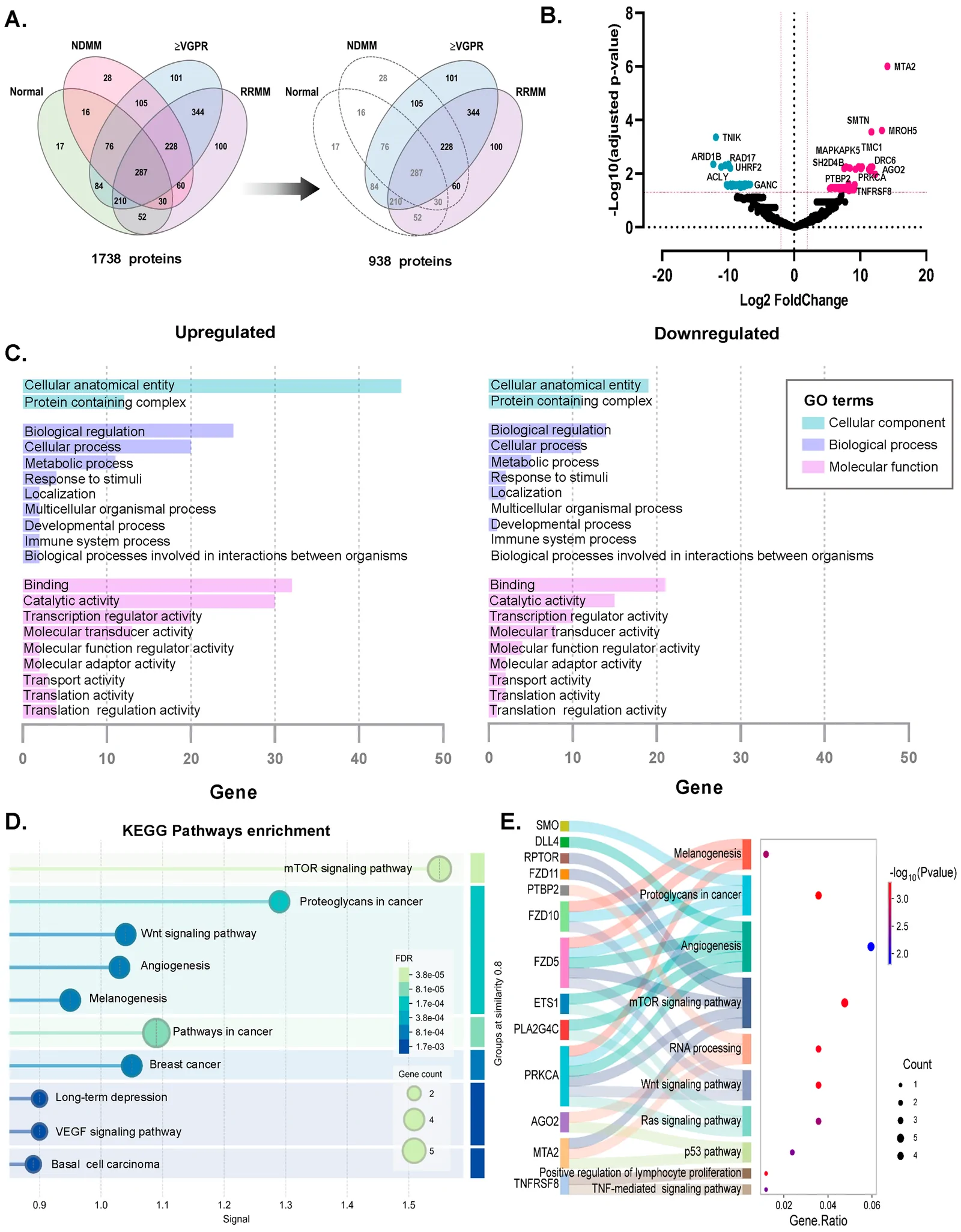
Proteomic profiling and functional enrichment of RRMM signature proteins. (**A**) Venn diagrams showing the overlap of proteins identified among normal controls, NDMM, ≥VGPR, and RRMM, with 1738 proteins initially detected and 938 proteins retained for downstream analysis. (**B**) Volcano plot of differentially expressed proteins in RRMM, showing log2 fold change vs. −log10 adjusted *p*-value. (**C**) Gene Ontology (GO) enrichment analysis of upregulated and downregulated RRMM signature proteins across cellular component, biological process, and molecular function categories. (**D**) KEGG pathway enrichment analysis of upregulated RRMM signature proteins, highlighting significantly enriched pathways. Dot size indicates gene count, and color represents the false discovery rate (FDR). (**E**) Sankey diagram and dot plot illustrating the relationships between key upregulated proteins and enriched KEGG pathways. The Sankey diagram was generated using SRplot [23] (available at https://www.bioinformatics.com.cn/en, accessed on 14 January 2026).

A serum proteomic dataset from our previous study identified 1738 proteins expressed across NDMM, ≥VGPR, RRMM, and normal control groups [18]. In the present study, 938 proteins expressed in the ≥VGPR and RRMM groups were selected for comparative analysis (Figure 2A). Among these proteins, 84 were significantly upregulated and 39 were significantly downregulated in the RRMM group compared with the ≥VGPR group, using fold-change thresholds of >1.5 and <−1.5, respectively (Figure 2B). All significantly differentially expressed proteins are listed in Supplementary Tables S1 and S2.

Among these RRMM signature proteins, the majority of both upregulated and downregulated proteins were significantly enriched in the GO category cellular anatomical entity. These proteins were mainly involved in biological regulation, as well as cellular and metabolic processes, and primarily functioned as binding, catalytic, and transcription regulator proteins (Figure 2C).

Based on KEGG pathway enrichment analysis, the upregulated proteins were significantly enriched in several physiological and pathological pathways, including the mechanistic target of rapamycin (mTOR) signaling pathway, proteoglycans in cancer, the Wnt signaling pathway, and angiogenesis (Figure 2D), whereas no significant pathway enrichment was observed among the downregulated proteins. The associations between key upregulated proteins and enriched KEGG pathways are shown in Figure 2E. Several proteins, including metastasis associated 1 family member 2 (MTA2), Argonaute 2 (AGO2), TNF receptor superfamily member 8 (TNFRSF8/CD30), protein kinase C alpha (PRKCA), Frizzled-5 (FZD5), and Frizzled-10 (FZD10), exhibited potential involvement in multiple pathways.

### 3.2. Analysis of Microarray Gene Expression Profiles in Myeloma Cells vs. Normal Plasma Cells

**Figure 3:**
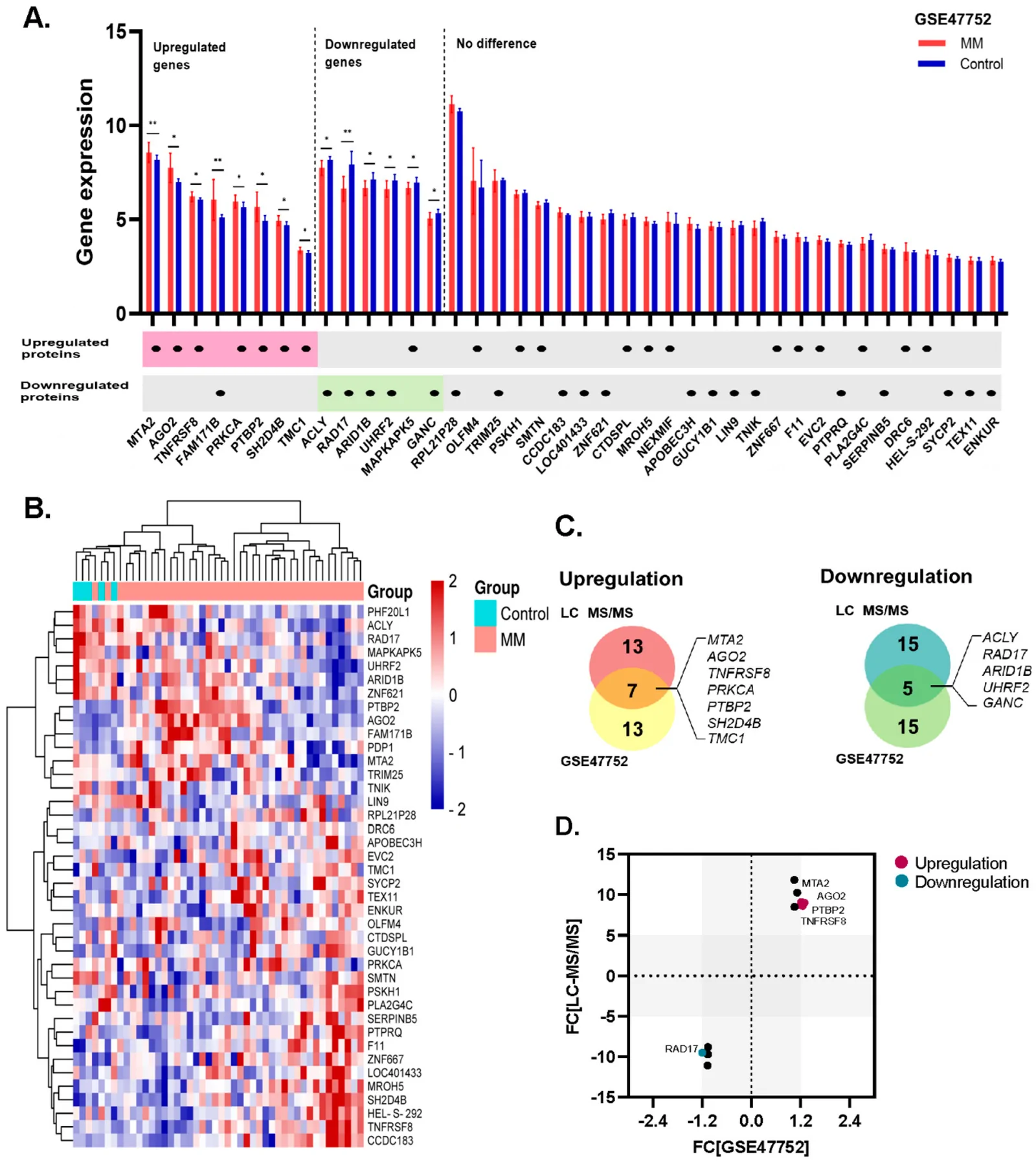
Integrated proteomic and gene expression analysis for biomarker identification. (**A**) Gene expression of the top 20 upregulated and top 20 downregulated proteins identified by LC–MS/MS, assessed in the GSE47552 microarray dataset, comparing myeloma cells with normal plasma cell controls. Statistical significance is indicated as follows: * denotes *p* < 0.05; ** denotes *p* < 0.01. (**B**) Heatmap with hierarchical clustering showing differential gene expression between MM and normal plasma cells. (**C**) Venn diagrams illustrating the overlap between differentially expressed proteins (LC–MS/MS) and genes (GSE47552 [20]) for upregulated and downregulated candidates. (**D**) Correlation of fold changes obtained from LC–MS/MS and GSE47552 analyses, with selected candidate biomarkers highlighted.

Publicly available microarray GEPs of myeloma cells and normal plasma cells (accession number GSE47552 [20]) obtained from the NCBI GEO were used to evaluate the potential associations of the top 20 upregulated and top 20 downregulated proteins in MM. Among the 40 genes encoding these proteins, only 14 genes were significantly differentially expressed between myeloma cells and normal plasma cells. Of these, eight genes—*MTA2*, *AGO2*, *TNFRSF8*, *FAM171B*, *PRKCA*, *PTBP2*, *SH2D4B*, and *TMC1*—were upregulated, whereas six genes—*ACLY*, *RAD17*, *ARID1B*, *UHRF2*, *MAPKAP5*, and *GANC*, were downregulated. The gene expression profiles comparing myeloma cells with normal plasma cells are illustrated in Figure 3A,B.

To evaluate concordant expression patterns, the overlap between differentially expressed proteins identified by LC–MS/MS and differentially expressed genes from the GSE47552 dataset [20] was analyzed for both upregulated and downregulated candidates. This analysis revealed that *MTA2*, *AGO2*, *TNFRSF8*, *PRKCA*, *PTBP2*, *SH2D4B*, and *TMC1* were consistently upregulated, whereas *ACLY*, *RAD17*, *ARID1B*, *UHRF2*, and *GANC* were consistently downregulated (Figure 3C).

### 3.3. Identification of Potential Serum Biomarkers

According to the aforementioned biomarker selection criteria, only five proteins—MTA2, AGO2, TNFRSF8, PTBP2, and RAD17—met the selection requirements (Figure 3D). As expected, MTA2 and AGO2 were significantly upregulated in disease-active states of MM, consistent with our previous report [18], and also showed significant differences in the present analysis. Therefore, these two previously validated proteins were not included for further investigation. In addition, the downregulated protein RAD17 was excluded because of its greater susceptibility to pre-analytical variation and biological confounding factors, such as consumption or dilution effects. Moreover, its relatively low abundance poses analytical challenges that may compromise reproducibility and clinical applicability. Consequently, only two upregulated proteins—TNFRSF8 and PTBP2—were selected as candidate biomarkers for further investigations.

### 3.4. Baseline Characteristics of 40 Relapsed MM Patients

The baseline characteristics of the 40 patients with relapsed MM included in this analysis are summarized in Table 1. Of these patients, 22 (55.0%) were male and 18 (45.0%) were female. The mean age (±SD) was 62.4 ± 10.1 years, and 27 patients (67.5%) were eligible for ASCT (age < 65 years). The majority of patients had an IgG isotype (70.0%), including 37.5% IgG kappa and 32.5% IgG lambda. Overall, 25 patients (62.5%) had ISS stage III disease at initial diagnosis. All patients received a bortezomib-based regimen as first-line therapy, subsequently achieved ≥VGPR, and were evaluated at their first documented relapse. None had primary refractory disease or a history of multiple relapses. The median follow-up duration was 74 months (range, 52–103 months).

### 3.5. Serum Concentrations of TNFRSF8 and PTBP2 in the Validation Cohort

**Figure 4:**
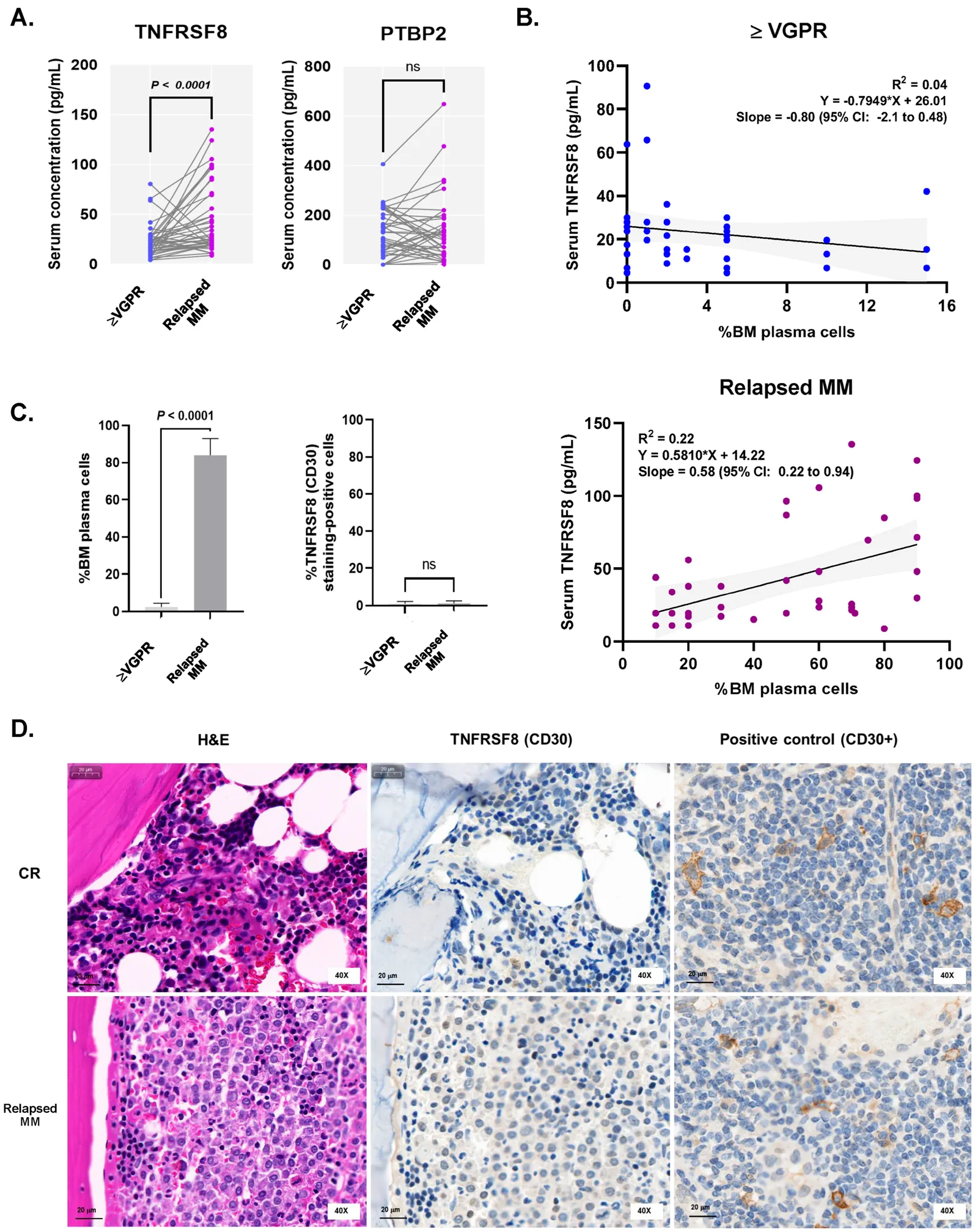
Evaluation of serum TNFRSF8 and its association with bone marrow plasma cell burden in relapsed MM. (**A**) Paired serum TNFRSF8 and PTBP2 concentrations at ≥VGPR and first relapse (*n* = 40 pairs). Measurements were performed in duplicate and compared using the Wilcoxon matched-pairs signed-rank test. (**B**) Linear regression analysis showing the correlation between serum TNFRSF8 levels and BMPC percentage at ≥VGPR and relapsed MM diagnosis (*n* = 40). (**C**) Paired BMPC and CD30-positive cell percentages at CR and relapse (*n* = 10 pairs). Comparisons were performed using a paired Student’s *t*-test, and data are presented as mean ± SD. (**D**) Representative H&E and CD30 IHC staining at CR and relapse (*n* = 10 pairs); tonsillar tissue served as a positive control.

In this validation phase, paired serum samples from 40 patients with relapsed MM, collected at the time patients first achieved a treatment response of ≥VGPR and at the time of relapsed MM diagnosis, were analyzed. Serum concentrations of TNFRSF8 and PTBP2 at ≥VGPR and at diagnosis of relapsed MM are shown in Figure 4A.

Serum TNFRSF8 levels at relapsed MM diagnosis were significantly higher than those at ≥VGPR, with median concentrations (IQR) of 31.9 (19.7–169.7) pg/mL and 20.61 (13.2–80.6) pg/mL, respectively (Wilcoxon signed-rank test, *p* < 0.001). The Hodges–Lehmann estimate of the paired difference was 12.67 pg/mL (95% CI, 6.37–20.33). (In contrast, no significant difference was observed in serum PTBP2 levels between relapsed MM diagnosis and ≥VGPR (median, 109.5 vs. 104.0 pg/mL; *p* = 0.578). Simple linear regression analysis was performed to assess the correlation between serum TNFRSF8 levels and the percentage of BM plasma cells. However, no significant linear correlation was observed at either ≥VGPR or at relapsed MM diagnosis, with *R*^2^ values of 0.04 and 0.22, respectively (*p* > 0.05; Figure 4B).

### 3.6. Expression of TNFRSF8 Detected by IHC in Bone Marrow Samples

To further validate the observed associations, IHC staining for TNFRSF8 (CD30) was performed on paraffin-embedded BM core biopsy samples from 10 patients with MM, collected at CR and at relapsed MM diagnosis (*n* = 10 per group). Consistent with our primary observations, no CD30-positive cells were detected in either the CR or relapsed MM groups, whereas the internal CD30-positive control derived from tonsillar tissue showed appropriate positive staining in selected cells. The percentage of BM plasma cells (BMPC) assessed by H&E staining differed significantly between the CR and relapsed MM groups, whereas no significant difference was observed in the percentage of CD30-positive cells between the two groups (Figure 4C,D).

## 4. Discussion

Relapse remains a major clinical challenge in MM, highlighting the importance of identifying circulating proteins associated with the relapse state. In this study, we reanalyzed a previously generated serum proteomic dataset, used an external transcriptomic dataset for candidate prioritization, and subsequently evaluated selected candidates by ELISA in an independent paired-serum cohort. Cross-platform concordance and subsequent candidate selection prioritized TNFRSF8 and PTBP2 for targeted ELISA evaluation. Of these, only TNFRSF8 showed a significant within-patient increase at first relapse compared with ≥VGPR, supporting its consideration as an exploratory circulating biomarker associated with the relapse state.

Using functional enrichment analyses, we demonstrated that proteins with increased abundance in RRMM compared with ≥VGPR were significantly enriched in pathways related to tumor progression and treatment resistance, including mTOR signaling, Wnt signaling, proteoglycans in cancer, and angiogenesis. These pathways are well recognized for their roles in MM pathogenesis, clonal evolution, and microenvironmental interactions that contribute to disease relapse [24,25,26]. The identification of TNFRSF8 among proteins involved in multiple enriched pathways suggests that it may reflect complex biological processes associated with RRMM rather than a single tumor-intrinsic mechanism.

Integration of proteomic and transcriptomic datasets represents a major strength of this study. Although 40 candidate proteins were initially evaluated, only 14 showed significantly differential gene expression in myeloma cells compared with normal plasma cells, supporting the use of cross-platform concordance for hypothesis-generating candidate prioritization. Further application of stringent fold-change thresholds reduced the candidate list to five proteins. After excluding previously evaluated biomarkers (MTA2 and AGO2) and proteins with limited analytical robustness (RAD17), TNFRSF8 and PTBP2 were selected for targeted ELISA evaluation. Notably, only TNFRSF8 demonstrated significant differences in serum levels between ≥VGPR and RRMM, supporting its prioritization as an exploratory relapse-associated circulating candidate.

TNFRSF8, also known as CD30, is a member of the tumor necrosis factor (TNF) receptor superfamily and is classically expressed in activated lymphocytes and certain lymphoid malignancies [27,28]. CD30 signaling can activate NF-κB through TNF receptor-associated factors (TRAFs) and engage mitogen-activated protein kinase pathways, including extracellular signal-regulated kinase (ERK), thereby modulating lymphoid-cell survival, proliferation, activation, and apoptosis in a context-dependent manner [28,29]. In germinal center B cells, CD30 expression has been shown to promote the expansion of IgG1-switched cells with elevated IgG1 levels [30], suggesting a role in B-cell activation and differentiation. Several studies have reported elevated expression of CD30 and its soluble form in various hematological malignancies, including classical Hodgkin lymphoma (HL), anaplastic large-cell lymphoma (ALCL), adult T-cell leukemia/lymphoma (ATLL), and acute myeloid leukemia (AML), and these elevations have been associated with poor clinical outcomes [31,32,33,34,35]. However, the role of CD30 in MM remains poorly defined, and studies investigating its clinical and biological significance in MM are limited.

In the present study, serum TNFRSF8 levels were significantly elevated at relapsed MM diagnosis compared with the ≥VGPR time point, whereas PTBP2 levels showed no significant change. Notably, serum TNFRSF8 levels did not correlate with BMPC burden, and TNFRSF8 expression was not detected in plasma cells by IHC in either CR or relapsed MM samples. Soluble CD30 is generally considered to be generated by proteolytic shedding of the extracellular domain of membrane-bound CD30. However, the ELISA used in this study measured circulating TNFRSF8 immunoreactivity and could not distinguish a shed extracellular fragment from other circulating forms or identify its cellular source. These findings suggest that circulating TNFRSF8 levels may not directly reflect tumor burden or plasma cell infiltration. This observation is consistent with previous reports indicating that CD30 expression in plasma cells is variable and often absent in malignant plasma cells [36]. Collectively, these findings raise the possibility that circulating TNFRSF8 may reflect alterations in the immune or BM microenvironment associated with disease progression or treatment resistance. However, the cellular sources and mechanisms underlying increased circulating TNFRSF8 remain to be determined.

TNFRSF8 expression is largely restricted to cells of the immune system and is expressed in activated T cells, B cells, and natural killer (NK) cells [37]. Based on the available literature, interactions between myeloma cells and the BM microenvironment may represent a plausible source of circulating TNFRSF8, or soluble CD30. Within the MM microenvironment, malignant plasma cells engage in dynamic interactions with immune effector cells, including T cells, B cells, and NK cells, creating a balance between immune surveillance and immune evasion [26]. Adhesion of myeloma cells to BM stromal cells induces the secretion of pro-inflammatory cytokines such as interleukin-6 (IL-6) and tumor necrosis factor-α (TNF-α), leading to sustained immune activation within the tumor niche [26,38]. Chronic tumor antigen exposure drives progressive CD8^+^ T-cell exhaustion, characterized by impaired cytotoxic function and the expression of inhibitory and activation markers, including programmed cell death protein 1 (PD-1) and TNFRSF8/CD30 [39,40]. Furthermore, MM progression is also associated with a shift in the immune microenvironment from a T helper 1 (Th1)-dominant, cell-mediated anti-tumor response toward a T helper 2 (Th2)-polarized, humoral and immunosuppressive immune profile. This immunological alteration is characterized by reduced cytotoxic T-cell and NK cell activity, increased production of regulatory and suppressive cytokines such as IL-4, IL-10, and IL-13, and an overall impairment of effective anti-myeloma cellular immunity, thereby supporting myeloma cell survival, proliferation, and escape from immune surveillance [41,42]. It has been reported that TNFRSF8 is preferentially expressed by human CD4^+^ T-cell clones that produce Th2-type cytokines and promotes the differentiation of T-cell lines toward a Th2-like profile [43]. Consequently, these biological processes may contribute to elevated circulating TNFRSF8 levels during MM disease progression and may explain why circulating TNFRSF8 levels are higher in relapsed MM than in remission states. However, further mechanistic studies are warranted to elucidate the cellular origin and biological role of circulating TNFRSF8 in MM.

Hypothetically, serum TNFRSF8 may represent a non-invasive and easily accessible biomarker that can be measured longitudinally, in contrast to cytogenetic and molecular risk factors that require BM sampling and are not routinely repeated during follow-up. Although MM may exhibit spatial heterogeneity, and a single-site BM examination may not fully represent disease involvement across all skeletal sites, BM assessment is only one component of the multimodal evaluation of treatment response. Established response assessment incorporates serum and urine monoclonal protein measurements, serum free light chains where applicable, BM findings, and imaging studies such as positron emission tomography/computed tomography (PET/CT) in selected settings. Circulating markers may provide complementary information by reflecting systemic disease-related processes; however, the present study did not directly compare TNFRSF8 with these conventional measures or evaluate whether TNFRSF8 changes preceded biochemical or clinical relapse. Its incremental value in disease monitoring therefore remains to be established in prospective longitudinal studies.

Several limitations of this study should be acknowledged. First, the validation cohort was relatively small and derived from a single center, which may limit the generalizability of the findings. Second, the validation cohort included only patients who had experienced relapse MM. Although paired serum samples demonstrated higher TNFRSF8 levels at relapse than at the ≥VGPR time point, this design does not allow assessment of whether TNFRSF8 can predict future relapse or distinguish patients who subsequently relapse from those who remain in response. Samples were obtained retrospectively from a local specimen bank according to clinical care and sample availability rather than a prospectively scheduled sampling protocol, limiting assessment of the temporal dynamics of TNFRSF8 during disease evolution. Moreover, conventional disease markers were not collected and analyzed according to a prespecified protocol at both time points; therefore, the diagnostic performance and incremental value of TNFRSF8 relative to established markers could not be determined. Third, the biological mechanisms and cellular sources underlying elevated circulating TNFRSF8 were not directly investigated. Although TNFRSF8 expression was not detected in plasma cells by IHC, this finding does not establish its cellular source. In addition, because TNFRSF8 is expressed by activated immune cells, its circulating concentration may be influenced by immune activation, concurrent infection, systemic inflammation, treatment-related immune changes, and renal dysfunction. Detailed information on these variables, including inflammatory biomarkers, renal function, and treatment exposure at each sampling time point, was not systematically collected. Consequently, these potential confounders could not be evaluated or adjusted for in this study. Fourth, TNFRSF8 levels across the full MM disease spectrum, including premalignant states such as monoclonal gammopathy of undetermined significance (MGUS) and smoldering MM (SMM), were not systematically evaluated. Finally, this study was designed as an exploratory biomarker investigation and was not intended to establish diagnostic accuracy, prognostic or predictive performance, or clinical utility. Larger, independent, prospective cohorts including both patients who subsequently relapse and those who remain in response, with comprehensive clinical and cytogenetic characterization and prespecified longitudinal sampling, are warranted to further investigate the clinical relevance and disease association of TNFRSF8.

## 5. Conclusions

In conclusion, serum TNFRSF8 concentrations were higher at first relapse than at ≥VGPR in paired samples. These findings support TNFRSF8 as an exploratory circulating candidate associated with the relapse state but do not establish its diagnostic, prognostic, or predictive utility, its incremental value over conventional MM markers, or its cellular origin. Prospective longitudinal studies incorporating non-relapsing comparators, serial pre-relapse sampling, and standardized concurrent assessment of established disease markers are required to determine its biological and potential clinical relevance.

## Figures and Tables

**Table 1:** Clinical characteristics of the 40 relapsed MM patients included in the validation cohort.

Characteristic		Relapsed MM Patients (*n* = 40)
**Age, year**		
	Mean (±SD)	62.4 (±10.1)
**Sex,** ***n*** **(%)**		
	Male	22 (55.0)
	Female	18 (45.0)
**Clonal isotype,** ***n*** **(%)**		
	IgG Kappa	15 (37.5)
	IgG Lambda	13 (32.5)
	IgA Kappa	5 (12.5)
	IgA Lambda	4 (10.0)
	Kappa light chain	2 (5.0)
	Lambda light chain	1 (2.5)
**Disease stage (ISS-stage),** ***n*** **(%)**		
	I or II	15 (37.5)
	III	25 (62.5)
**Transplant eligibility,** ***n*** **(%)**		
	Eligible (age < 65 year)	27 (67.5)
	Non-eligible (age ≥ 65 year)	13 (32.5)
**Patient receiving ASCT,** ***n*** **(%)**		
	Yes	25 (62.5)
	No	15 (37.5)
**First line treatment regimen,** ***n*** **(%)**		
	Bortezomib + Cyclophosphamide + Dexamethasone (VCD)	19 (47.5)
	Bortezomib + Melphalan + Prednisone (VMP)	12 (30.0)
	Bortezomib + Thalidomide + Dexamethasone (VTD)	9 (22.5)
Overall follow-up, months *		
	Median (IQR)	74 (57.5–96.3)
	Range	52–103

* Overall follow-up was counted from the date of the initial diagnosis. ASCT, autologous stem cells transplantation; Ig, immunoglobulin; ISS, the International Staging System; IQR; interquartile range, SD, standard deviation.

## Data Availability

The original contributions presented in this study are included in the article/Supplementary Material. Further inquiries can be directed to the corresponding author.

## Supplementary Materials

The following supporting information can be downloaded at: https://www.mdpi.com/article/10.3390/diseases14100380/s1, This study includes Supplementary Tables S1 and S2, and raw pictures for CD30 IHC staining, which are provided in the Supporting Information file. Supplementary Table S1: Significantly upregulated proteins identified between the RRMM and ≥VGPR groups. Supplementary Table S2: Significantly downregulated proteins identified between the RRMM and ≥VGPR groups.

## Abbreviations

The following abbreviations are used in this manuscript:

AGO2	Argonaute 2
ASCT	Autologous stem cell transplantation
BMPC	Bone marrow plasma cells
CAR	Chimeric antigen receptor
CR	Complete response
GEPs	Gene expression profiles
H&E	Hematoxylin and Eosin
IHC	Immunohistochemistry
IMWG	International Myeloma Working Group
ISS	International Staging System
LC–MS/MS	Liquid chromatography–tandem mass spectrometry
MDEs	Myeloma-defining events
MM	Multiple myeloma
MR	Minimal response
MTA2	Metastasis associated 1 family member 2
PTBP2	Polypyrimidine tract-binding protein 2
RRMM	Relapsed and/or refractory multiple myeloma
TNFRSF8	Tumor necrosis factor receptor superfamily member 8
VGPR	Very good partial response
