# Supplementary material for: Exploratory Identification of Potential Serum Biomarkers Associated with Relapse in Multiple Myeloma
Source: Diseases. 2026 Oct 16;14(10):380. doi: 10.3390/diseases14100380 (PMC13703590; doi:10.3390/diseases14100380)

Supporting Information file:

Exploratory Identification of Potential Serum Biomarkers Associated with Relapse in Multiple Myeloma

*Wittawat Chantkran [1], Chonlada Laoruangroj [2], Sittiruk Roytrakul [3], Oytip Nathalang [4], Pasra Arnutti [5], Janeyuth Chaisakul [6], and Dollapak Apipongrat [1, 2,] **

1 Department of Pathology, Phramongkutklao College of Medicine, Bangkok 10400, Thailand; chantkran@yahoo.com (W.C.); dollapak.a@gmail.com (D.A.)

2 Division of Hematology, Department of Medicine, Phramongkutklao Hospital, Bangkok 10400, Thailand; amchon25@gmail.com (C.L.)

3 Proteomics Research Laboratory, National Center for Genetic Engineering and Biotechnology, Pathumthani 12120, Thailand; sittiruk@biotec.or.th (S.R.)

4 Graduate Program in Biomedical Sciences, Faculty of Allied Health Sciences, Thammasat University, Pathumthani 12120, Thailand; oytipntl@hotmail.com (O.N.)

5 Department of Medical Technology, Faculty of Allied Health Sciences, Pathumthani University, Pathumthani12000, Thailand; pasra@hotmail.com (P.A)

6 Department of Pharmacology, Phramongkutklao College of Medicine, Bangkok 10400, Thailand; janeyuth.cha@pcm.ac.th (J.C.)

* Correspondence: dollapak.a@gmail.com

Supplemental documents:

- **Table S1** Significantly upregulated proteins identified between the RRMM and ≥VGPR groups
- **Table S2** Significantly downregulated proteins identified between the RRMM and ≥VGPR groups
- **Raw Pictures for CD30 IHC staining**

Table S1 Significantly upregulated proteins identified between the RRMM and ≥VGPR groups

No.	Accession no.	Gene name	Protein name	Peptide sequence	Log2FC	*P*-value
1	A0A024R534	*MTA2*	Metastasis associated 1 family, member 2	AECSIRLPKAAK	14.14	9.30E-05
2	A0A087WYG4	*MROH5*	Maestro heat-like repeat family member 5	ACWEEQLIQMAIKSVPFLSTDVWSK	13.29	2.40E-04
3	Q8NEE6	*DRC6*	Dynein regulatory complex subunit 6	AAAEESNFPER	12.28	6.88E-04
4	Q8TDI8	*TMC1*	Transmembrane channel-like protein 1	ESLRPKRK	11.84	1.07E-03
5	B3KY41	*SMTN*	Smoothelin	ENWLHSQQREAEQR	11.71	1.21E-03
6	B4DF67	*MAPKAPK5*	MAPK-activated protein kinase 5	AKSCGERDVK	11.56	1.40E-03
7	X6R3B1	*F11*	Coagulation factor XI	EIESQNLIGSYRVLK	11.5	1.49E-03
8	A0A0C4DGE7	*EVC2*	Ellis van Creveld syndrome 2	ADADLEACR	11.33	1.75E-03
9	Q9Y3X7	*DKFZ*	Uncharacterized protein DKFZp586C1620	LCHDNNEDHQLDRFK	10.27	4.53E-03
10	Q9ULA1	*ZNF667*	Zinc finger protein 667	CNKCEKVCNR	10.13	5.13E-03
11	A0A0K0K1J0	*HEL-S-292*	Epididymis secretory sperm binding protein	DGQCPLFPFTERK	9.77	6.92E-03
12	A0A024R2L8	*CTDSPL*	Carboxy-terminal domain, RNA polymerase II, polypeptide A, small phosphatase-like	ARLFRESCVFHR	9.31	1.01E-02
13	P11801	*PSKH1*	Protein serine kinase H1	GNYQQQR	9.18	1.12E-02
14	P28908	*TNFRSF8*	Tumor necrosis factor receptor superfamily member 8 (CD30L receptor)	CTACVSCSRDDLVEKTPCAWNSSR	9.12	1.17E-02
15	A0A2R8YEQ5	*NEXMIF*	Neurite extension and migration factor	EQVHKDESGTASFEK	9	1.29E-02
16	B4DI28	*PTBP2*	Polypyrimidine tract-binding protein 2	IDFSKLVNLNVK	8.73	1.58E-02
17	A0A024QZ95	*OLFM4*	Olfactomedin 4	ELQIVKLEMK	8.65	1.68E-02
18	Q9UKV8	*AGO2*	Argonaute2	AALAQHRDGNSQGSTHSGEASDHK	8.64	1.70E-02
19	A0A024QZH0	*PLA2G4C*	Phospholipase A2, group IVC	QEWDLAKSLQKTIQAAR	8.62	3.73E-02
20	Q5SQS7	*SH2D4B*	SH2 domain-containing protein 4B	GQIRALSHRHYER	8.5	1.88E-02
21	A0A169TED2	*PRKCA*	Protein kinase C	ECMGFDAK	8.48	1.91E-02
22	A0A0A0MQS0	*PHF20L1*	PHD finger protein 20-like protein 1	IDYEEGKMLVHFER	8.449	1.95E-02
23	Q495X7	*TRIM60*	Tripartite motif-containing protein 60	CLNQKYHHMK	8.414	2.00E-02
24	A0A024R2I4	*C3orf20*	Chromosome 3 open reading frame 20	AMVESMSVGANPLDITR	8.336	2.12E-02
25	A0A024R3F5	*MIZF*	MBD2-interacting zinc finger	EHEDGYMRLQLVR	8.306	2.17E-02
26	Q9BY89	*KIAA1671*	Uncharacterized protein KIAA1671	RTVPLKDRPR	8.288	2.20E-02
27	B7Z1N6	*ALDOA*	Fructose-bisphosphate aldolase	AEVNGLAAQGK	8.26	2.24E-02
28	Q8N7U5	*EPHA5*	Receptor protein-tyrosine kinase	IPIRWTAPEAIAFR	8.229	2.29E-02

29	A0A0C4DG02	*DNMT3A*	DNA (cytosine-5-)-methyltransferase 3A	DISRFLESNPVMIDAK	8.228	2.30E-02
30	Q6ZP01	*RBM44*	RNA-binding protein 44	ACFTTSR	8.164	2.40E-02
31	Q6PFW1	*PPIP5K1*	Inositol hexakisphosphate and diphosphoinositol-pentakisphosphate kinase 1	GVLSPGRHVRTR	8.129	2.46E-02
32	Q58EY0	*RORB*	RAR related orphan receptor B isoform 1	KVQKLQEK	8.118	2.48E-02
33	Q2NL82	*TSR1*	Pre-rRNA-processing protein TSR1 homolog	QLPGRPHAAM	7.949	2.80E-02
34	Q12899	*TRIM26*	Tripartite motif-containing protein 26	LLAWVGDERPLSLDKRK	7.923	2.85E-02
35	Q8NB16	*MLKL*	Mixed lineage kinase domain-like protein	ETLRQYLPPK	7.871	2.96E-02
36	Q6ZNE1	*EIF3A*	Eukaryotic translation initiation factor 3 subunit A	DRPDLSAPESLQLYLDTR	7.858	2.98E-02
37	F8W7T1	*DPF3*	Zinc finger protein DPF3	EYFQDGRLTCMHFLNEK	7.853	2.99E-02
38	Q9P2L0	*WDR35*	WD repeat-containing protein 35	IADEEAKKGSKPLR	7.815	3.08E-02
39	O14917	*PCDH17*	Protocadherin-17	GGGGLQPHSYEARICNYR	7.808	3.09E-02
40	Q9Y2H9	*MAST1*	Microtubule-associated serine/threonine-protein kinase 1	APRFKEYVHNMMVIENDYSSPR	7.764	3.19E-02
41	A0A024R9R5	*ZFP106*	Zinc finger protein 106 homolog	DIWNSTEQNPLETSR	7.751	3.71E-02
42	J3KN75	*TBC1D8B*	TBC1 domain family member 8B	EDDPEKFR	7.732	3.52E-02
43	F5H450	*FZD10*	Frizzled-10	ADVHRAGLYPHPR	7.726	1.14E-02
44	P08575	*PTPRC*	Receptor-type tyrosine-protein phosphatase C	CPDYIIQKLNIVNKK	7.656	3.47E-02
45	U3KQG0	*TARS2*	Threonyl-tRNA synthetase	AHILVRPELAGAQK	7.644	3.36E-02
46	Q13467	*FZD5*	Frizzled-5	GRPCCRCSTRR	7.576	6.32E-03
47	Q9ULX3	*NOB1*	RNA-binding protein NOB1	TFCGHRCLIYR	7.568	3.35E-02
48	Q86XN6	*ZNF761*	Zinc finger protein 761	CEECDKAFHFK	7.562	3.82E-02
49	F8W9B8	*EXOC5*	Exocyst complex component 5	GYSHCVDVYIK	7.519	3.54E-02
50	Q7L5A3	*FAM214B*	Protein FAM214B	GLREPGETSPAR	7.483	3.36E-02
51	A0A024R4Z5	*GLYATL2*	Glycine N-acyltransferase-like protein	LEEVLSYSNVISWEQTLQIQGCQEGLDEAIRK	7.464	3.92E-02
52	Q8N122	*RPTOR*	Regulatory-associated protein of mTOR	DSICFWDWEK	7.445	3.32E-02
53	B3KVF0	*ERCC3*	DNA helicase	DIISHESVQSGIAR	7.392	3.74E-02
54	A0A2R8Y6J6	*SCN10A*	Sodium channel protein 10A	MMVCEGTFVAVFFQNK	7.378	3.33E-02
55	Q5SXM1	*ZNF678*	Zinc finger protein 678	AGCEECPYKPEGSHK	7.378	3.60E-02
56	A0A024RAC0	*LUZP1*	Leucine zipper protein 1	AANGLEADNSCPNSK	7.259	3.78E-02
57	Q96GD3	*SCMH1*	Polycomb protein SCMH1	GAFDYWCRFDSR	7.182	6.25E-04
58	J3KP02	*LEKR1*	Leucine-, glutamate- and lysine-rich protein 1	EAQSLAETLERFEECR	7.177	3.33E-02
59	A5PLL1	*ANKRD34B*	Ankyrin repeat domain-containing protein 34B	AELCAHMLAKTGK	7.131	3.68E-02

60	Q9NXH8	*TOR4A*	Torsin-4A	EFAVTGCKQVVATVNLL	7.105	4.95E-02
61	Q9GZY0	*NXF2*	Nuclear RNA export factor 2	DVSYKIYDDENQK	7.095	8.13E-02
62	B2R825	*B2R825*	Alpha-1,4 glucan phosphorylase	CDYYHQQR	7.079	3.37E-02
63	A8K9S3	*A8K9S3*	Coronin	ETDLNWIMK	7.032	7.46E-02
64	Q9ULJ7	*ANR50*	Ankyrin repeat domain-containing protein 50	EHGMYAAAILAR	7.021	3.33E-02
65	D3DTW0	*D3DTW0*	Tubulin tyrosine ligase-like family, member 6	ERLLEQILRQAYER	7.015	3.61E-02
66	Q14CC5	*TPH2*	Tryptophan 5-monooxygenase	ELSKLYPTHACR	6.91	3.60E-02
67	Q14692	*BMS1*	Ribosome biogenesis protein BMS1 homolog	EVKEEIDPDEEESAKK	6.907	3.50E-02
68	F8WBB6	*F8WBB6*	Dipeptidyl-peptidase IV	QYLYEHDSIRWLSYK	6.906	3.73E-02
69	B9EIS5	*CM1*	PCM1 protein	ALNVPPSLDCR	6.886	3.45E-02
70	X5D7N2	*PRODH*	Proline dehydrogenase	HALLAPWACRLLVLLR	6.886	3.42E-02
71	Q86Y64	*ZNF354A*	Zinc finger protein 354A	CFKLEGSHKNEK	6.866	3.58E-02
72	A0A087X254	*ZNF615*	Zinc finger protein 615	ICSGRGAPGGQPEIR	6.854	3.38E-02
73	A0A2R8YG28	*OTOA*	Otoancorin	ISPIEIGLFISYDNATK	6.843	3.33E-02
74	C9J1J2	*NME6*	Nucleoside diphosphate kinase 6	AIPGSAMFEVR	6.736	1.13E-02
75	B5M0C0	*DUOXA1*	Dual oxidase maturation factor 1	AARRDYDWK	6.587	1.13E-02
76	Q9UKT9	*IKZF3*	Zinc finger protein Aiolos	AEMSNGAPQELEKK	6.582	3.32E-02
77	F8VZA0	*PUS7L*	Pseudouridylate synthase 7 homolog-like protein	DKKAITYQAMVVR	6.579	3.43E-02
78	Q7Z6I6	*RHG30*	Rho GTPase-activating protein 30	AYFRELPDPLLTYR	6.567	3.35E-02
79	Q9NR61	*DLL4*	Delta-like protein 4	KELEVDCGLDKSNCGK	6.545	3.38E-02
80	Q6FG54	*ETS1*	ETS1 protein	GLSFENVAWMVWR	6.518	1.09E-02
81	Q5JWV1	*UCKL1*	Uridine-cytidine kinase-like 1	ALAPEMTEGR	6.508	3.45E-02
82	C9JBE8	*MXD1*	Max dimerization protein 1	GKLELCRLLHR	6.487	1.09E-02
83	Q6ICI8	*APOL4*	Apolipoprotein L4	GHKVEIENAIR	6.393	3.45E-02
84	V9HWF1	*HEL-S-305*	Kelch-like protein 15	KPLLLKISNVMSHGIMR	6.391	3.33E-02

Table S2 Significantly downregulated proteins identified between the RRMM and ≥VGPR groups.

No.	Accession no.	Gene name	Protein name	Peptide sequence	Log2FC	P-value
1	A0A090N7Y3	LOC401433	Uncharacterized LOC401433	QIGAGEPAETAPCTVLRVR	-12.24	7.14E-04
2	Q9UKE5	TNIK	TRAF2 and NCK-interacting protein kinase	ENEEHKRQLLAER	-11.88	1.02E-03
3	Q96PU4	UHRF2	E3 ubiquitin-protein ligase UHRF2	KCEPDGGCLDCEPDRR	-11.06	2.23E-03
4	B7TQM8	APOBEC3H	Apolipoprotein B mRNA editing enzyme catalytic subunit 3H	AQGRYMDILCDAEV	-10.43	3.95E-03
5	M0R181	RPL21	60S ribosomal protein L21	CHPMKGQVTGMK	-10.23	4.70E-03
6	B4DWS9	SERPINB5	Serpin B5	FSESETKECPFRVNK	-10.06	5.43E-03
7	A0A087WZU1	PTPRQ	Protein-tyrosine-phosphatase	DPASELTTVIMEASK	-9.956	5.92E-03
8	B4DYX8	PDPC1	Pyruvate dehydrogenase phosphatase 1	CCCRCGM	-9.842	6.52E-03
9	Q8IYF3	TEX11	Testis-expressed protein 11	DKSSWYGHEHFVPK	-9.7	7.34E-03
10	Q4LE36	ACLY	ATP-citrate (pro-S-)-lyase	GLRILVGAAPL	-9.689	7.40E-03
11	A0A1B0GV63	ARID1B	AT-rich interactive domain-containing protein 1B	ICEFAFLYQIYK	-9.684	7.43E-03
12	A0A087WUX1	ENKUR	Enkurin	ALNEQIRYDYDEQKK	-9.64	7.71E-03
13	A0A0G2JPT5	RAD17	Cell cycle checkpoint protein RAD17	EDTLAEKMRLGFR	-9.483	8.76E-03
14	Q9BX26	SYCP2	Synaptonemal complex protein 2	DEFNELEEIIIFQKFDK	-9.258	1.05E-02
15	Q6ZSS3	ZNF621	Zinc finger protein 621	AFDFHQSVRNLLGGVPR	-9.248	1.06E-02
16	Q5T5S1	CCDC183	Coiled-coil domain-containing protein 183	FSTIDWVHSRCVAKSK	-9.167	1.13E-02
17	Q6P995	FAM171B	Protein FAM171B	ALLVVALGLLLTCPR	-8.901	1.39E-02
18	Q02153	GUCY1B1	Guanylate cyclase soluble subunit beta-1	EIDEWVEPGYR	-8.785	1.52E-02
19	Q6P142	LIN9	LIN9 protein	CSSAFFEEERSALK	-8.775	1.53E-02
20	Q59GW5	TRIM25	Tripartite motif-containing 25	DEFEFLEKASK	-8.704	1.61E-02
21	Q2M2A3	GANC	Glucosidase, alpha neutral C	DCNKIAFYRR	-8.639	1.69E-02
22	B7ZAX3	PDE4DIP	Phosphodiesterase 4D interacting protein	CEEYKDIIDSVLR	-8.612	1.73E-02
23	Q8TC76	FAM110B	Protein FAM110B	VHSKVQSREAK	-8.601	1.74E-02
24	B3KMW2	UPS36	Ubiquitin carboxyl-terminal hydrolase 36	KVLTWDGKMSAVSQDAIEDSR	-8.424	1.99E-02
25	A0A024R1I0	NF2, RNF207	Neurofibromin 2	ESVDHDLVECNFEMEADMTVR	-8.329	2.13E-02
26	A8K9I1	STK11	Serine/threonine protein kinase11	CIERDEVRNVFR	-8.274	2.22E-02
27	F8W7T7	ZNF44	Zinc finger protein 44	ETIRNLNCIGMK	-8.256	2.25E-02

28	B4DIF6	TM6SF1	Transmembrane 6 superfamily member 1	CIYKPEFFIKTK	-8.182	2.37E-02
29	A1L447	USP29	Ubiquitin carboxyl-terminal hydrolase 29	EDNPVPNKK	-8.182	2.37E-02
31	P51160	PDE6C	cGMP phosphodiesterase 6C	CGIRLFFEINVVEKFK	-8.098	2.52E-02
32	C9J069	AJM1	Apical junction component 1 homolog	ALANELHPIK	-8.062	2.58E-02
33	A0A0C4DG08	ZNF780	Zinc finger protein 780A	ECEFKPEGTHKTQHQSLQLYRLFK	-8.05	2.61E-02
34	A0A6Q8PH34	ZEB2	Zinc finger E-box-binding homeobox 2	DITEPDTDMK	-8.037	2.63E-02
35	A0A024QZZ4	ZNF322A	Zinc finger protein 322A	KVHVIFARSR	-7.973	2.75E-02
36	Q96K83	ZNF521	Zinc finger protein 521	EHITQIHNQLESESEK	-7.91	2.88E-02
37	L8E8Y4	C8orf4	Alternative protein C8orf4	DLKTLPSLQER	-7.689	3.35E-02
38	B3KX52	TMEM132B	Transmembrane protein 132B	CESKVIVDEDTK	-7.636	3.48E-02
39	H3BRF9	ZFYVE19	Abscission/NoCut checkpoint regulator	CAGCDGDLFCARCFR	-7.615	3.53E-02

Raw Pictures for IHC

RRMM No.2864: H&E

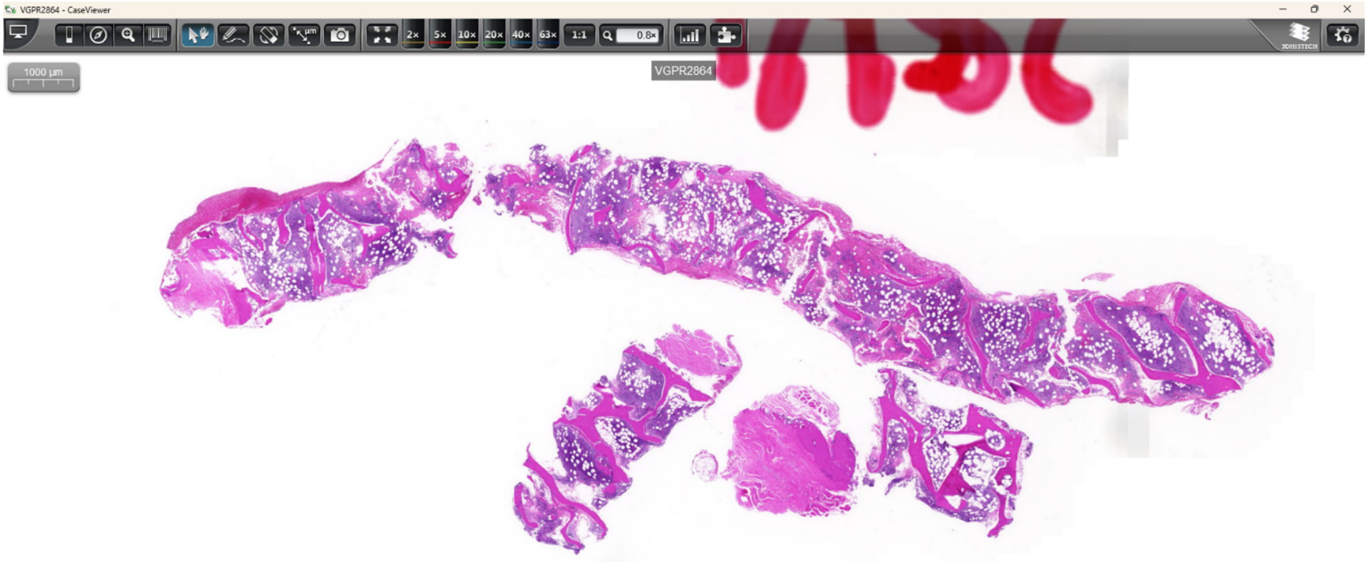

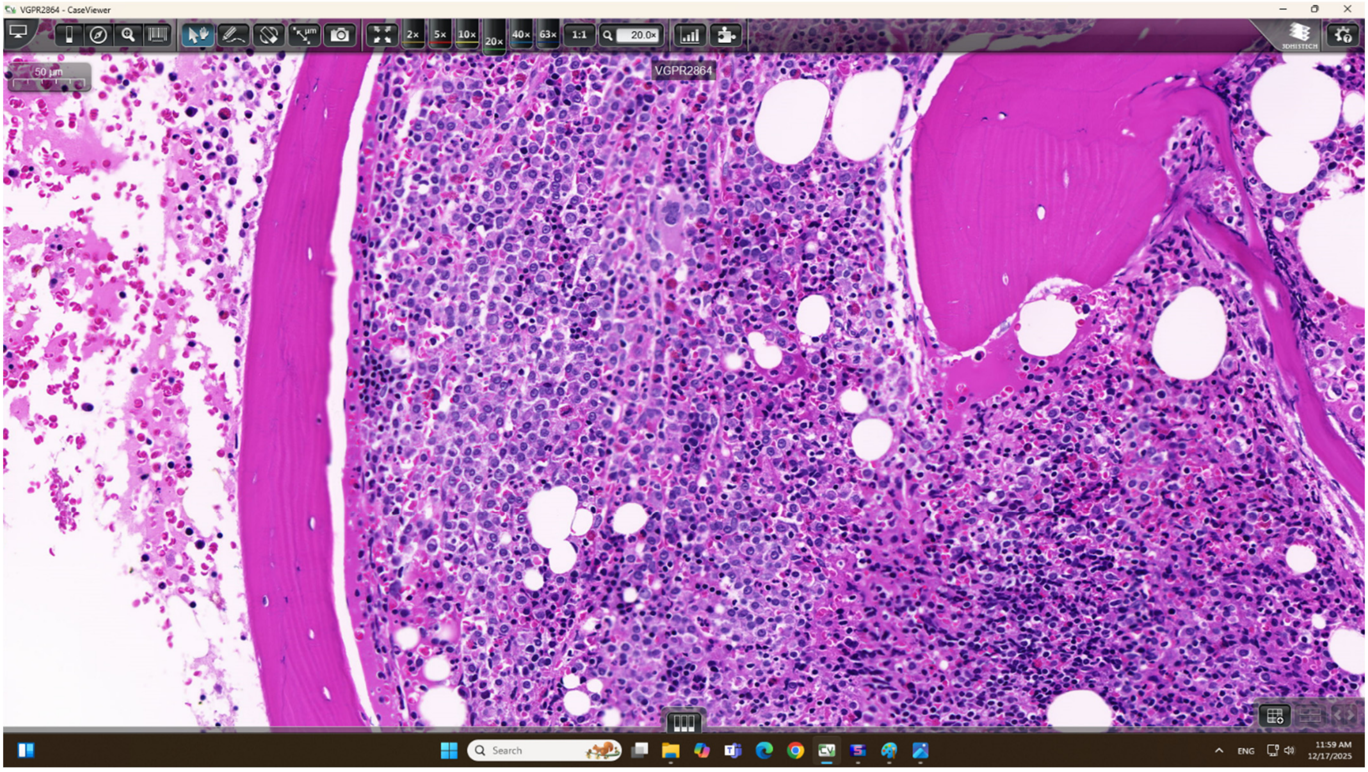

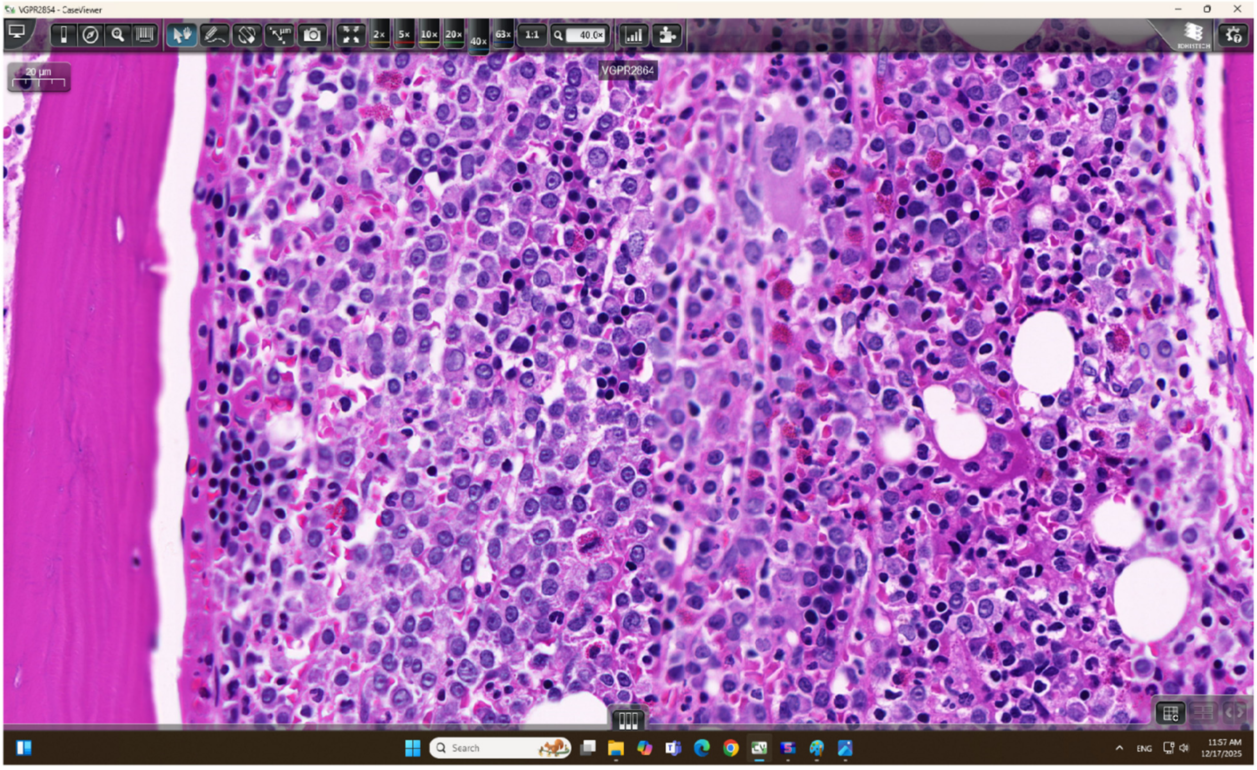

RRMM No.2864: CD30

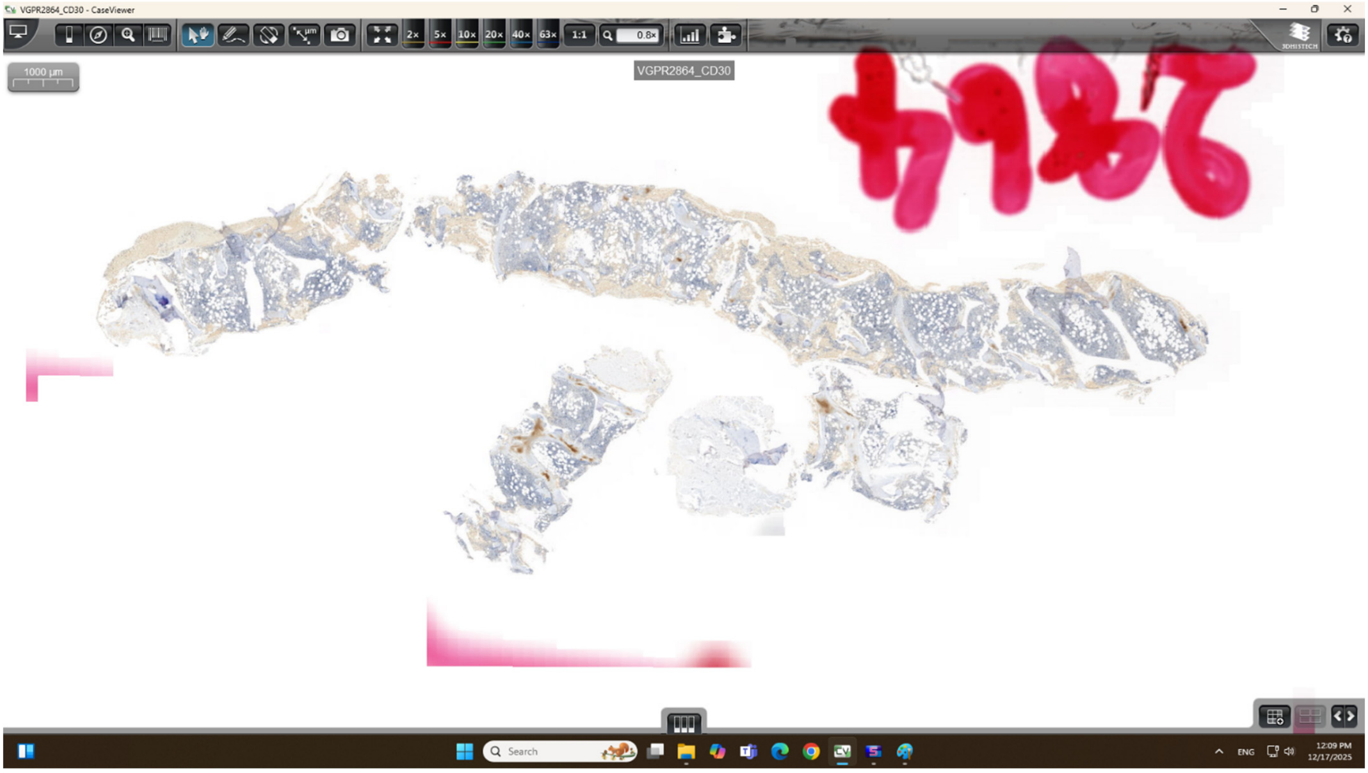

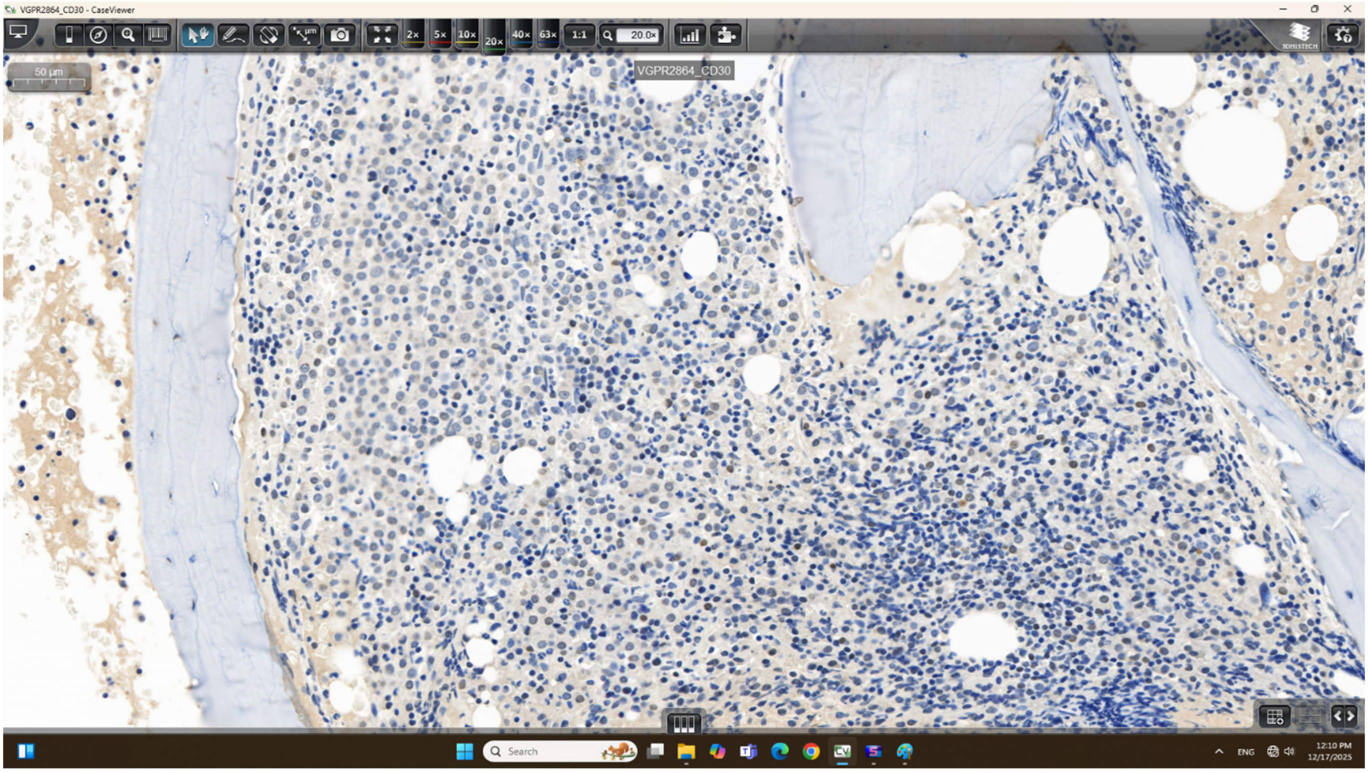
VGPR2864_CD30 - CaseViewer
2x 5x 10x 20x 40x 63x 1:1 20.0x
50 µm
VGPR2864_CD30
Search
ENG
12:10 PM
12/17/2025

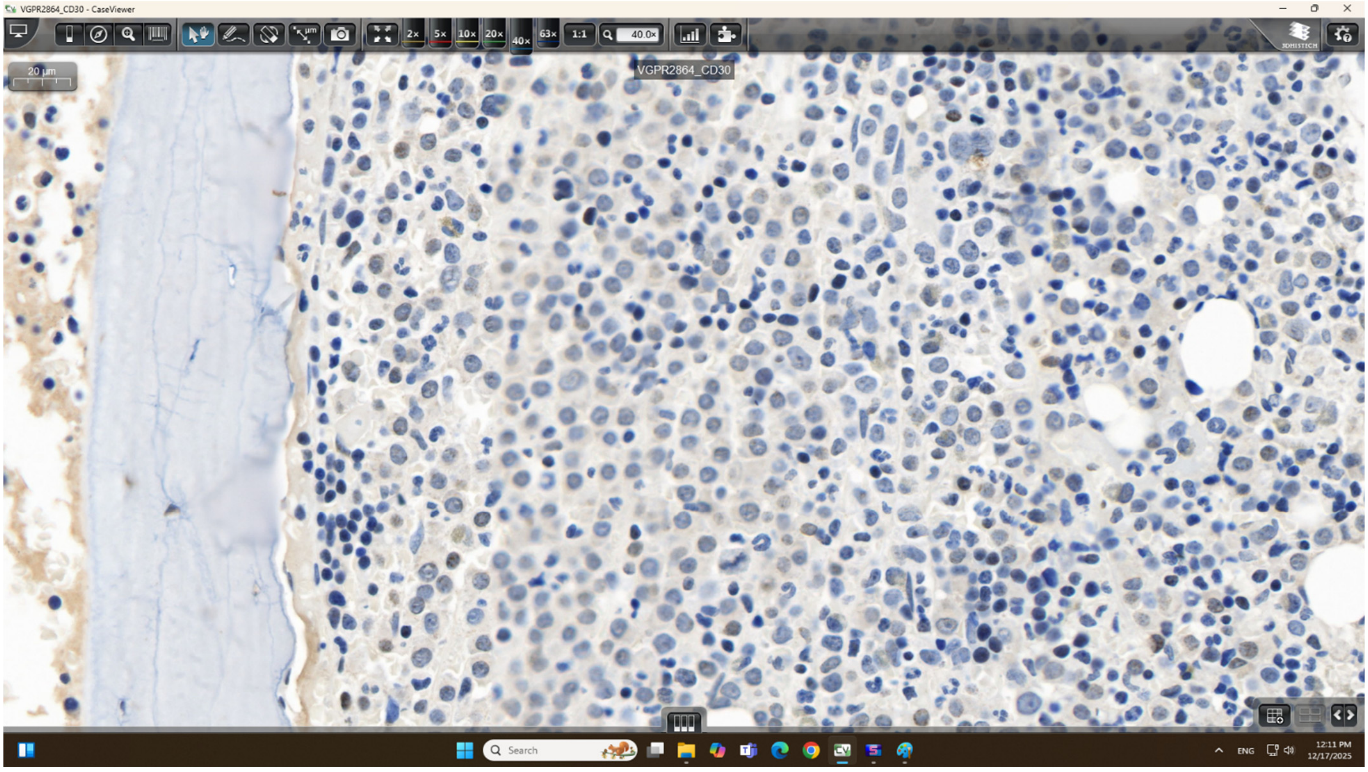
VGPR2864_CD30 - CaseViewer
2x 5x 10x 20x 40x 63x 1:1 40.0x
20 µm
VGPR2864_CD30
Search
ENG
12:11 PM
12/17/2025

Internal CD30-positive control

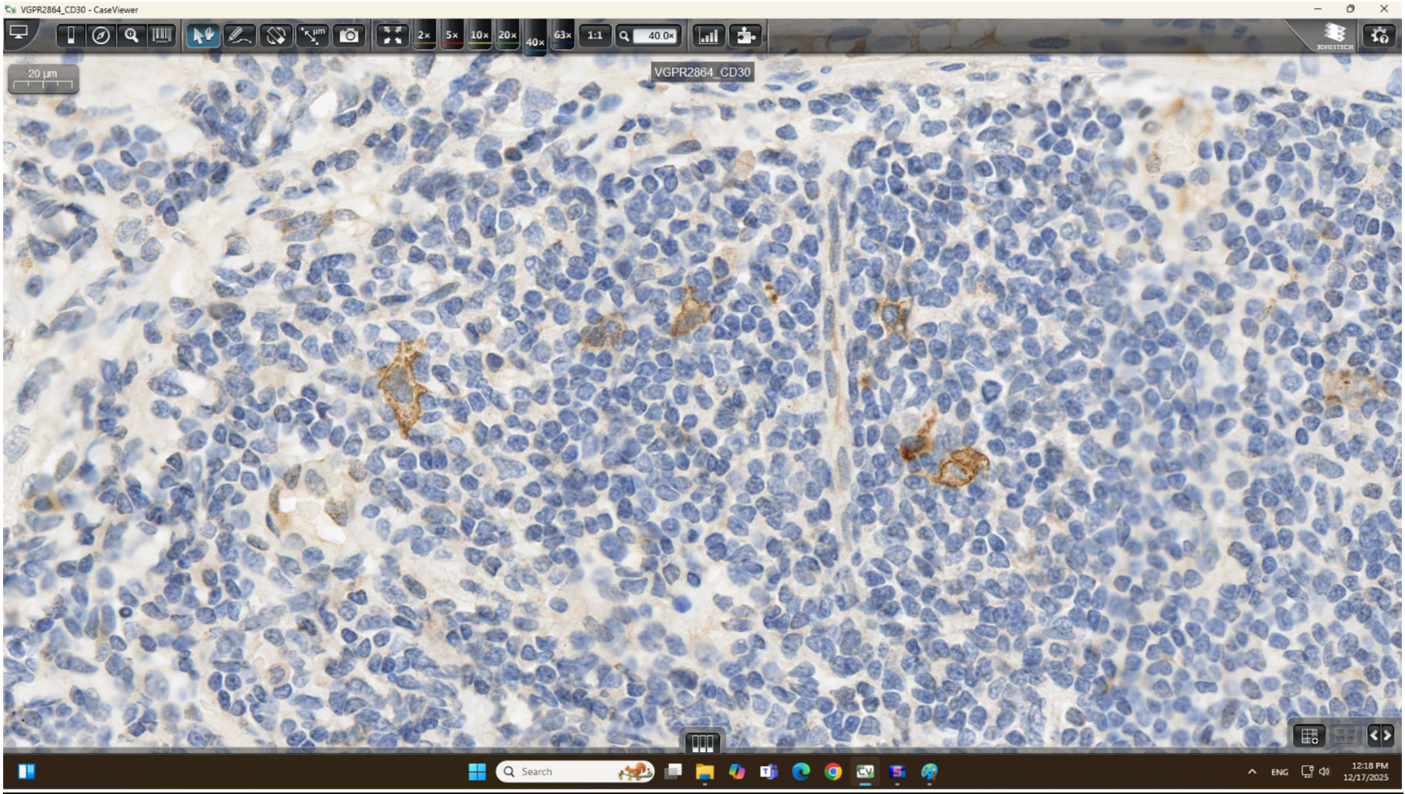

Raw Pictures for IHC

CR No.9579: H&E

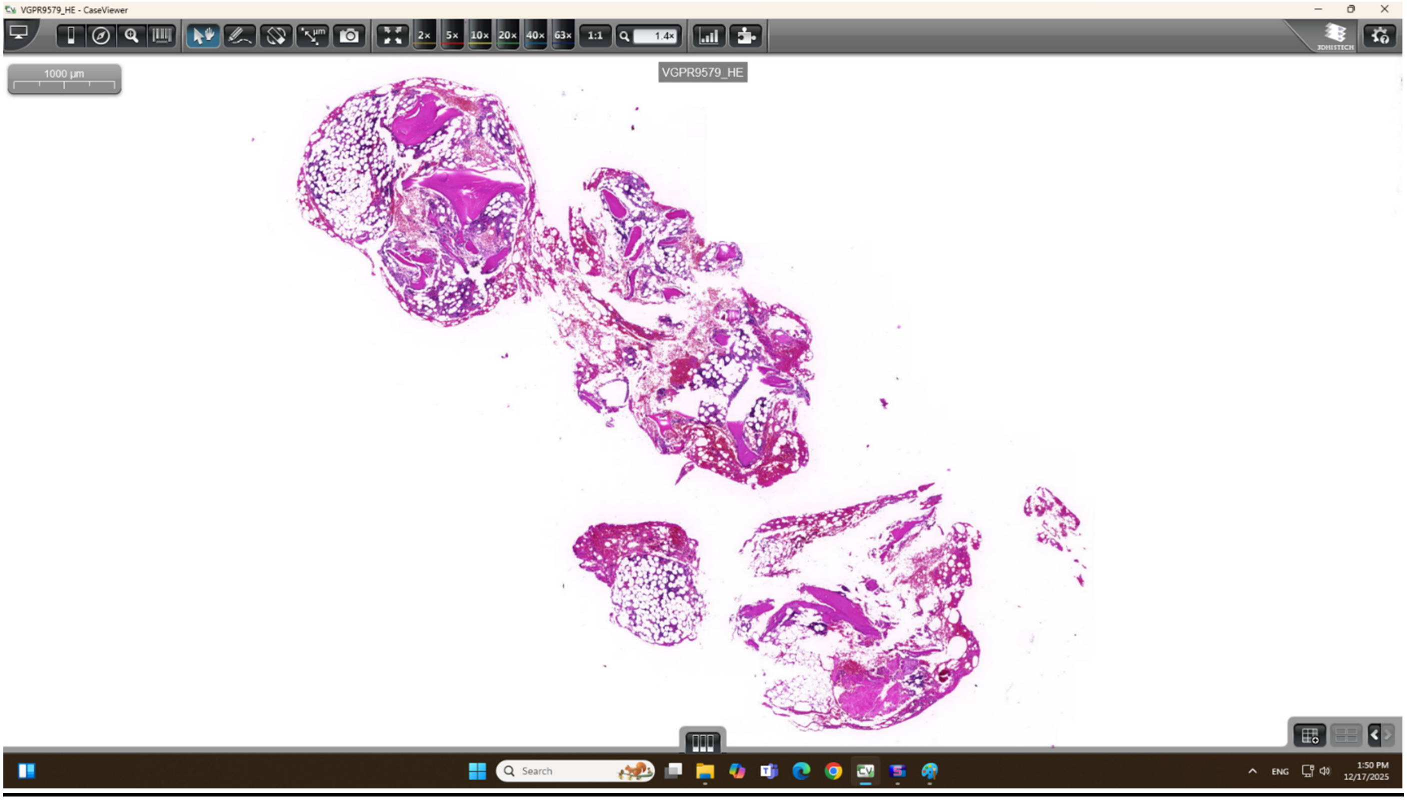

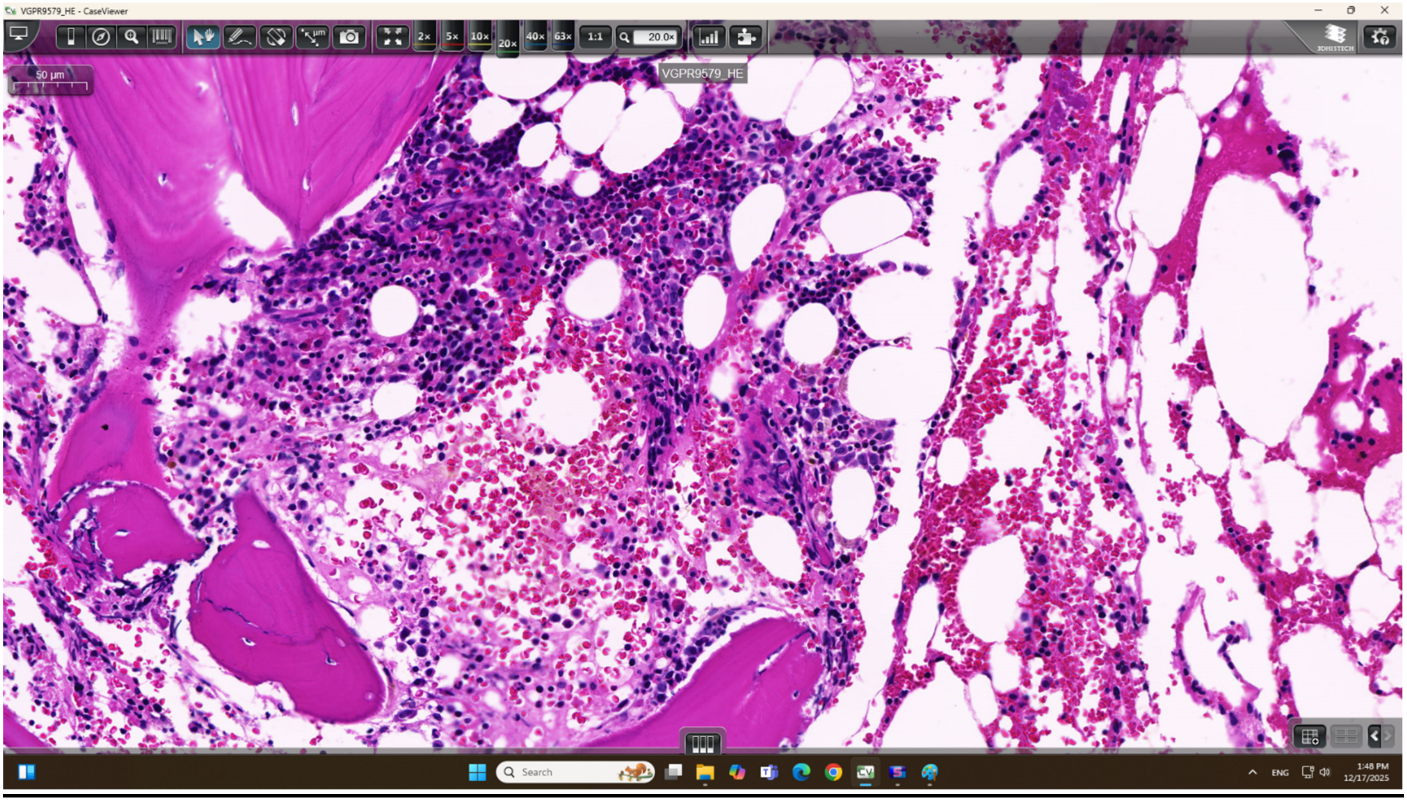

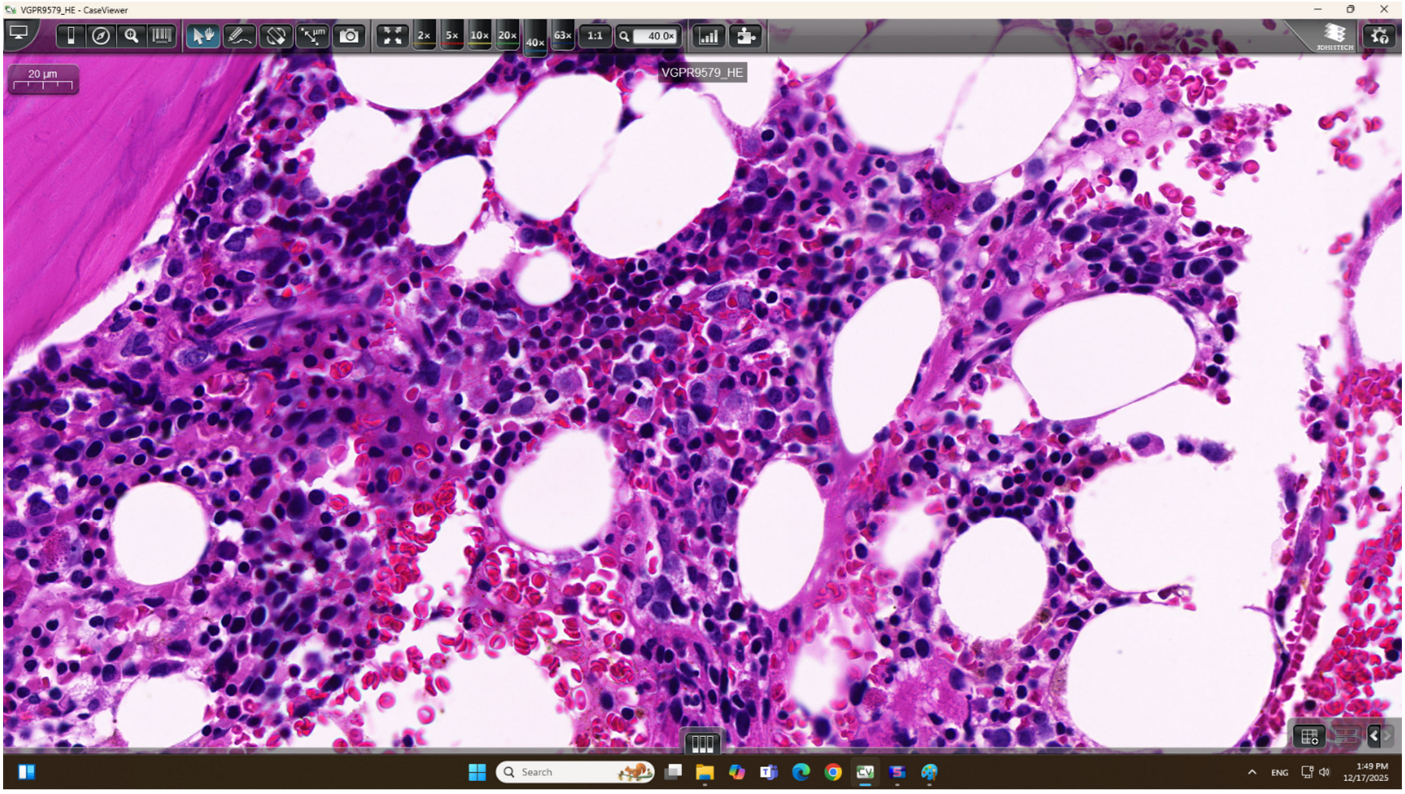

CR No.9579: CD30

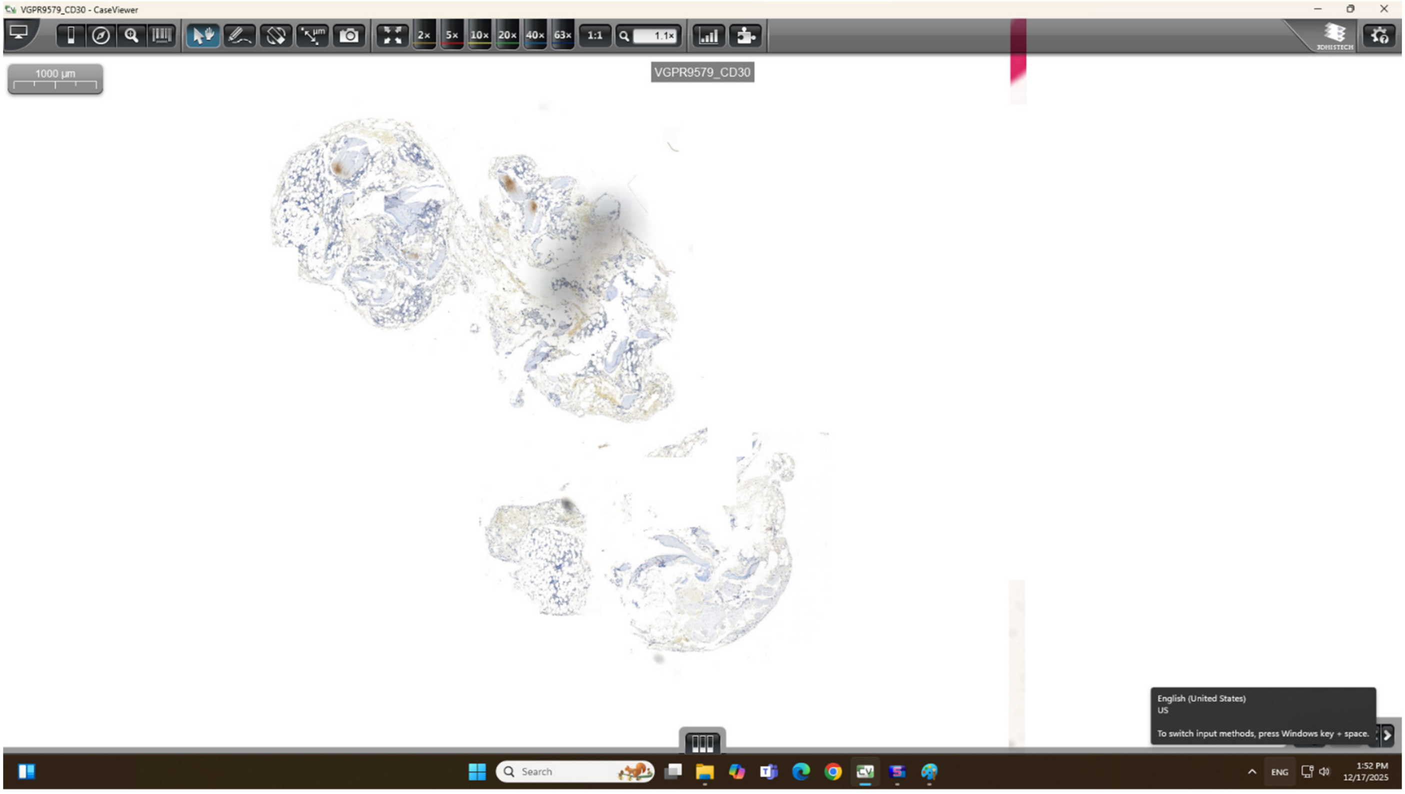

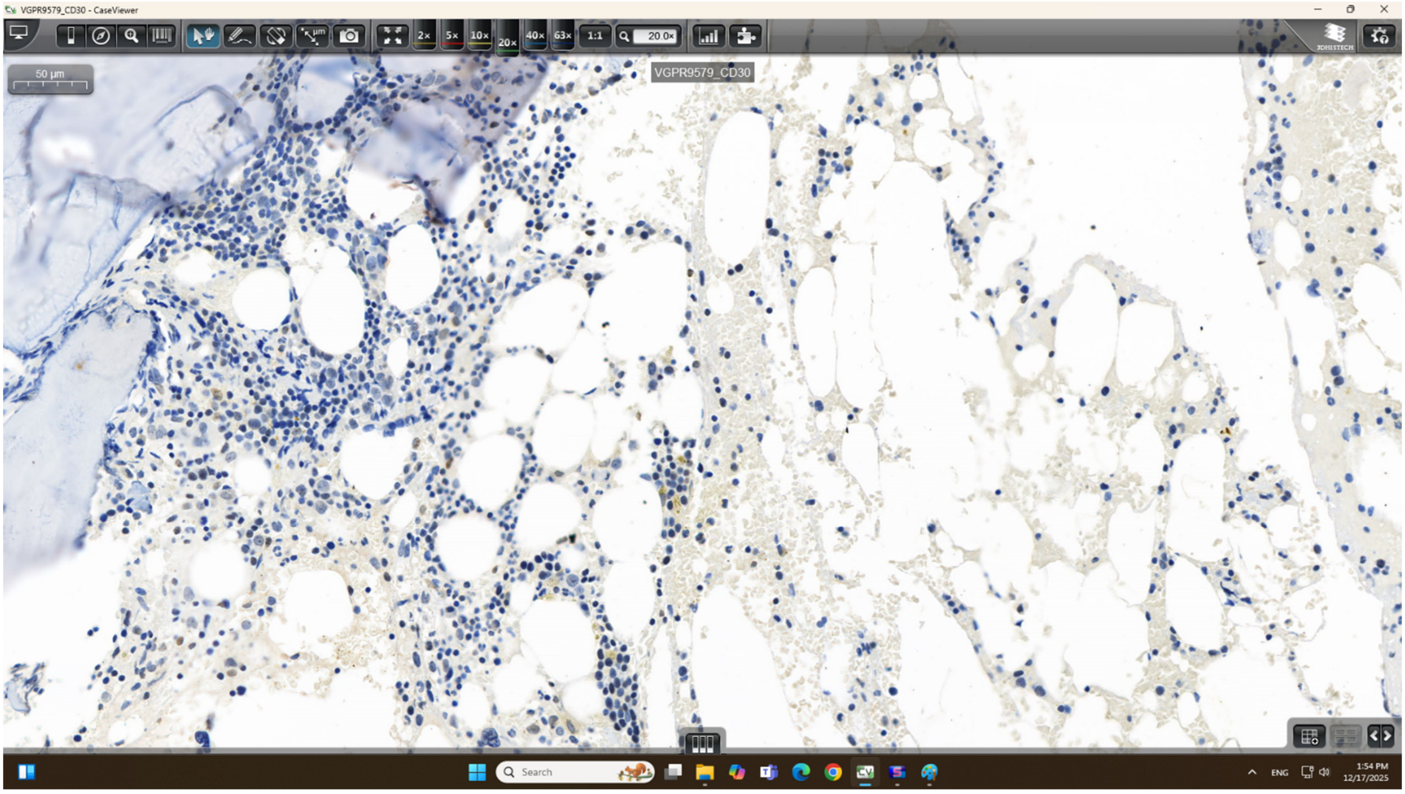
VGPR9579_CD30 - CaseViewer
VGPR9579_CD30
50 µm

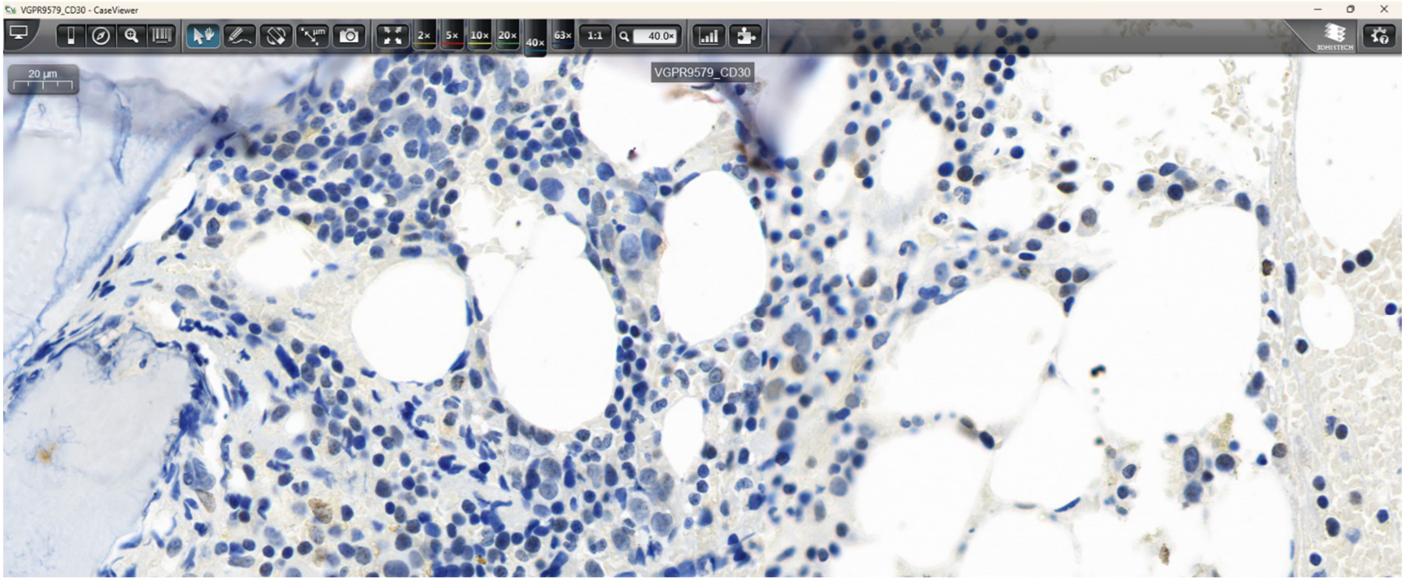
VGPR9579_CD30 - CaseViewer
VGPR9579_CD30
20 µm

Internal CD30-positive control

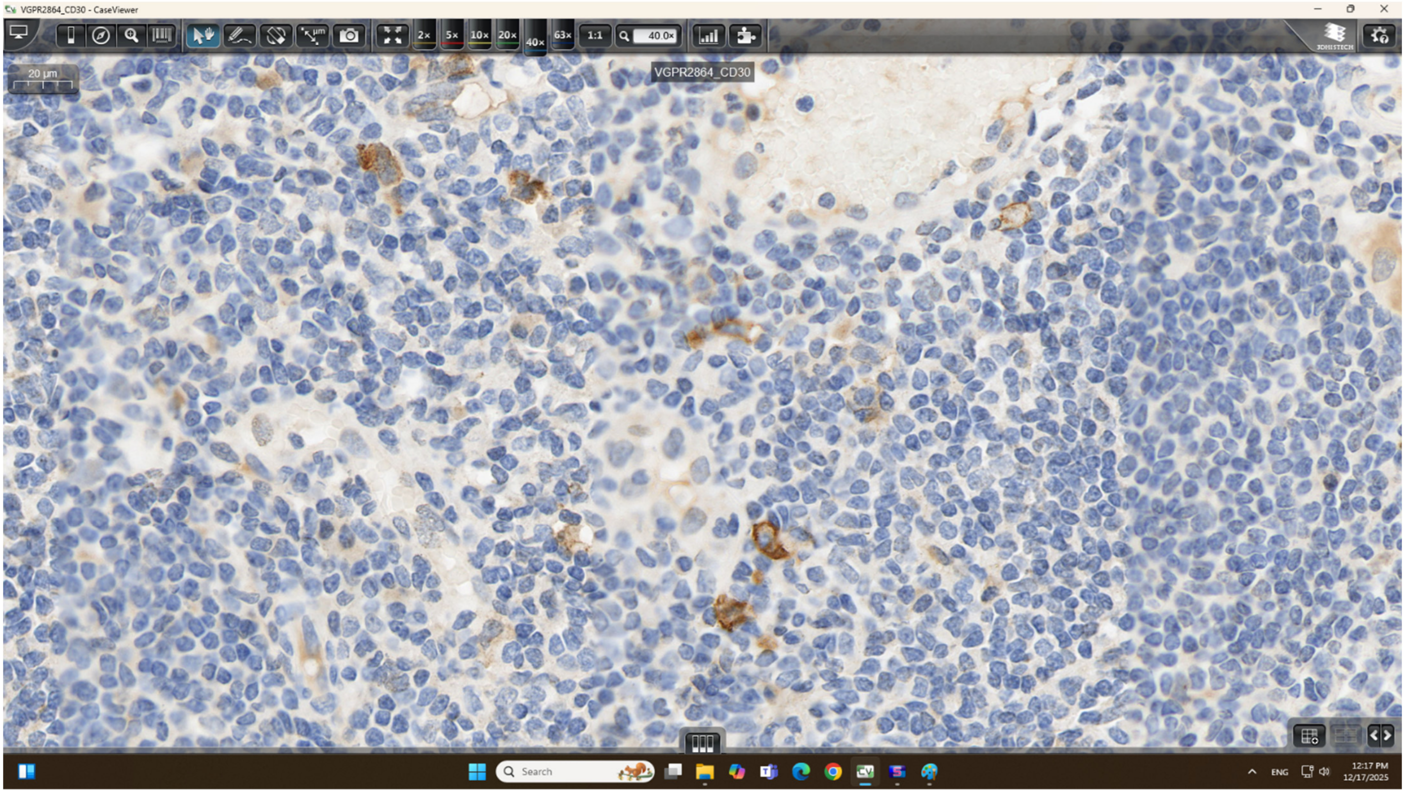

Supplement: Supplementary file 1 [file diseases-14-00380-s001.zip › diseases-4545303-supplementary.pdf]
